# Impact of Sensory Deficits on Upper Limb Motor Performance in Individuals with Cerebral Palsy: A Systematic Review

**DOI:** 10.3390/brainsci11060744

**Published:** 2021-06-03

**Authors:** Isabelle Poitras, Ophélie Martinie, Maxime T. Robert, Alexandre Campeau-Lecours, Catherine Mercier

**Affiliations:** 1 Center for Interdisciplinary Research in Rehabilitation and Social Integration, Quebec City, QC G1M 2S8, Canada; isabelle.poitras.2@ulaval.ca (I.P.); ophelie.martinie.1@ulaval.ca (O.M.); maxime.robert@fmed.ulaval.ca (M.T.R.); alexandre.campeau-lecours@gmc.ulaval.ca (A.C.-L.); 2 Department of Rehabilitation, Laval University, Quebec City, QC G1V 0A6, Canada; 3 Department of Mechanical Engineering, Laval University, Quebec City, QC G1V 0A6, Canada

**Keywords:** cerebral palsy, upper extremity, motor skills, sensations

## Abstract

People living with cerebral palsy (CP) exhibit motor and sensory impairments that affect unimanual and bimanual functions. The importance of sensory functions for motor control is well known, but the association between motor and sensory functions remains unclear in people living with CP. The objective of this systematic review was to characterize the relationship between sensory deficits and upper limb motor function in individuals living with CP. Methods: Five databases were screened. The inclusion criteria were: (1) including people living with CP, (2) reporting measurements of upper limb motor and sensory functions. A qualitative analysis of the studies’ level of evidence was done. Results: Thirty-three articles were included. Twenty-five articles evaluated tactile functions, 10 proprioceptive functions and 7 visual functions; 31 of the articles reported on unimanual functions and 17 of them reported on bimanual functions. Tactile functions showed a moderate to high association; it was not possible to reach definitive conclusions for proprioceptive and visual functions. Conclusions: The heterogeneity of the results limits the ability to draw definitive conclusions. Further studies should aim to perform more comprehensive assessments of motor and sensory functions, to determine the relative contribution of various sensory modalities to simple and more complex motor functions.

## 1. Introduction

Performing upper limb movements, such as reaching for a glass and bringing it to one’s mouth, requires the integration of sensory information from various modalities (e.g., tactile, proprioceptive, and visual). In the case of this example, vision provides information about the distance between the hand and the glass to grasp, as well as about the characteristics of the glass (e.g., shape and texture) [1] which makes it possible to choose the appropriate hand aperture [2] while predicting the required grip and load force [3]. Touch provides information about the surface of the glass and whether sufficient pressure is being exerted to prevent it from slipping [4]. Proprioception provides information about the movement and the position of the hand and the mouth [5]. The example given here illustrates how sensory processing is essential to the production of motor commands that are adapted to the environment based on sensorimotor experience, as is acknowledged by contemporary theories of motor control [6,7,8,9], and is supported by observations of individuals who have complete or partial sensory deprivation. Deafferented patients who have a complete loss of proprioceptive inputs exhibit slower movements and more restricted movement patterns [5], for example, which can lead to an inability to perform some motor tasks. It has also been shown that an alteration in sensory processing among elderly people [10] leads to slower reaction times, a higher risk of fall [11], and an overall reduction in motor performance [12,13]. These examples highlight the importance of assessing sensory functions in populations with neurological disorders, in particular in the case of people who have motor deficits.

Cerebral palsy (CP) is a group of disorders mainly characterized by posture and movement deficits [14] including spasticity [15], limited range of motion, muscle weakness [16] and lack of motor coordination, combined with sensory deficits [17]. It is clear that the atypical development of the corticospinal tract contributes to these motor deficits, since a contralateral development of the corticospinal tract (as compared with what is observed in typically developing children [18]) leads to lesser deficits than ipsilateral development, or than mixed lateralization [19,20]. However, the thalamocortical tract, one of the main sensory pathways, is also frequently damaged in individuals living with CP, which yields the possibility of substantial sensory deficits [21]. In fact, over 50% of people living with CP report sensory disorders [17] in addition to motor disorders, including tactile [22], proprioceptive [23] and visual deficits [24]. Tactile deficits encompass deficits in detection of mechanical stimuli, in two-point discrimination, texture recognition, graphesthesia, and stereognosis [25,26]. Proprioceptive deficits include deficits in joint-position sense and kinesthesia [27] while visual deficits regroup ophthalmological abnormalities (e.g., strabismus), cerebral visual impairment (e.g., binocular visual acuity) and visual perception deficits (e.g., visual orientation [28]). Nevertheless, while it has received increasing attention in scientific literature over the past two decades [26], the assessment of sensory deficits is not used systematically and extensively during rehabilitation, which might lead to misconceptions about the causes of motor performance difficulties.

Although hemiparetic CP is the most frequent type of CP [29], motor deficits are typically observed in both limbs, with one being less affected than the other [30]. Deficits in tactile and proprioceptive functions have also been shown to be present in both upper limbs [27], and visual deficits present in individuals with CP [24,31] may also impact on the motor performance of either limb. However, the extent to which deficits in each of these sensory modalities influence upper limb motor function remains unclear. In particular, it is still unknown whether sensory deficits could have a greater impact on bilateral motor functions (BMF) than on unimanual motor functions (UMF), given the increased need for complex sensorimotor integration in BMF.

The aim of this systematic review is therefore to characterize the relationship between sensory deficits (including tactile, proprioceptive and visual functions) and upper limbs motor functions (including UMF and BMF) in individuals with CP.

## 2. Materials and Methods

This systematic review follows the Preferred Reporting Items for Systematic Reviews and Meta-Analyses (PRISMA) [32] and was registered with the International Prospective Register of Systematic Reviews (CRD42020161016) on 3 December 2019 (access to the protocol: PROSPERO—International prospective register of systematic reviews. Available online: https://www.crd.york.ac.uk/prospero/display_record.php?RecordID=161016 (accessed on 1 June 2021).

### 2.1. Data Sources

Five databases were screened: CINAHL, Medline, Web of Science, Embase, and PsycINFO. Systematic weekly alerts [33] for each database were programmed to keep the review up to date (from the original database screening on 22 November 2019 until 15 December 2020). The databases were systematically searched by combining general keywords and specialized terms specific to each database. Keywords were organized around three different concepts: (1) cerebral palsy; (2) somatosensory and visual functions; and (3) upper limb motor performance (unilateral and bilateral) (see Supplementary Materials, Table S1).

To be included in the review, articles had to: (1) include individuals diagnosed with CP, regardless of the individuals’ age or CP type (hemiparesis, quadriparesis, and diplegia); (2) report clinical or kinematic measurements of upper limb motor functions (UMF or BMF) and clinical assessments of sensory functions (visual, touch, proprioception); (3) present empirical data; and (4) be published through a peer-reviewed process. More specifically for the main outcomes (sensory and motor functions), articles had to meet at least one of three criteria: they had to (1) present associations between motor and sensory variables; (2) compare the motor performance of a group of individuals with CP who have sensory deficits, to that of individuals with CP who do not have sensory deficits; or (3) provide raw individual data that would make one of these analyses possible. This approach was used to include a maximum number of relevant articles. Studies reporting assessment of pain or temperature threshold have been excluded. Case studies, book chapters, meta-analyses, systematic reviews, conference abstracts, and articles written in languages other than English or French were also excluded. Titles and abstracts were screened independently by two reviewers (I.P. and O.M.) to identify their eligibility, and then, the selected articles’ full text was screened by these same reviewers to determine their eligibility. Article selection was discussed until consensus was reached and any disagreement was resolved by a third reviewer (C.M.).

### 2.2. Data Extraction

One author (I.P.) performed the data extraction for the 33 selected articles and then the second author (O.M.) corroborated the extracted results. Variables extracted were based on a standardized tool (see Supplementary Files, Table S2) (Better systematic review management. Available online: www.covidence.org (accessed on 1 June 2021) and consisted of: (1) participants’ characteristics (level of impairment, age, sex); (2) type of CP; (3) timing of lesion; (4) type of lesion; (5) sensory assessments; (6) sensory outcomes; (7) sensory modality (tactile, visual, proprioception); (8) motor assessments; (9) motor outcomes; (10) type of motor functions (UMF or BMF); and (11) description of assessments and results.

Two types of study design were retrieved from the screening: cross-sectional studies with correlational or regression analysis, and interventional designs with raw data of the baseline outcomes. Results extracted from the cross-sectional studies were correlation coefficient (r, Pearson or Spearman), square coefficient (r^2^), Beta coefficient (β), coefficient of regression, and percentage based on regression model. For the interventional studies, raw data from the baseline were extracted and correlation analysis (Pearson or Spearman correlations) were performed using IBM SPSS Statistics for Windows, Version 26.0 (Armonk, NY, USA: IBM Corp.). Results were considered significant when *p* < 0.05. Correlation coefficient were ranked as follows: perfect = +/−1.0, strong = +/−0.7–0.99; moderate = +/−0.4–0.69; low = +/−0.1–0.39 and no correlation = <+/−0.1 [34]. When other types of results were reported (such as β coefficient, regression models or group comparison) conclusions were adjusted accordingly.

#### 2.2.1. Descriptions of Sensory Functions

Sensory functions were categorized into three modalities: tactile, proprioceptive, and visual. For each modality, various functions were assessed:

1. Tactile Functions: Processing of sensory inputs arising from different types of receptors located in the skin to distinguish texture, vibration, shape, or pressure (pain and temperature were not included in this review). Tactile functions were generally separated into two main categories: registration (stimulus detection) and perception (spatial, temporal, and modality-specific characteristics) [35]. In this review, tactile functions have been subcategorized through a combination of these definitions and the tactile assessments performed:
  - Tactile registration—Tactile pressure detection: Force required to bend the thinnest Semmes-Weinstein monofilament detected by the participant when applied on the fingers, the palm, or the back of the hand.
  - Tactile registration—Tactile vibration detection: Identification of the finger stimulated with a vibration applied on a fingertip.
  - Tactile perception—Two-point discrimination: Capacity to discriminate two pressure points applied on a fingertip. Assessed using an esthesiometer by identifying the smallest distance between two pressure points detected by the participant.
  - Tactile perception—Stereognosis: Identification of different usual or abstract objects when manipulated in the hand, without vision, with and without moving or the fingers.
  - Tactile perception—Graphesthesia: Identification of numbers or letters drawn on the back or the palm of the hand.
  - Tactile perception—Directionality: Capacity to identify the direction of tactile stimulation performed on a part of the body (e.g., top-down).
  - Tactile perception—Location of stimulus: Identification of the specific location of a single stimulus applied on a part of the body.
  - Tactile perception—Double simultaneous stimulation: Capacity to identify the part of the hand touched, when one stimulus is applied to both sides.
  - Tactile perception—Temporal discrimination: Capacity to identify the timing at which a stimulus occurred. In this review, this refers to a temporal order judgement between two stimuli applied on the fingers.
  - Tactile perception—Texture discrimination: Capacity to distinguish different types of texture, such as silky, soft, or rough.
2. Proprioceptive functions: Perception of upper limb position and movement, sense of tension, force, effort, or balance [36]. More specifically, this has been characterized in this systematic review as the capacity to either detect or reproduce a movement or a position (at the wrist, elbow or shoulder joint) when actively moved by the subject or when passively moved by an examiner/robot.
3. Visual functions: Ability to see with the eyes (encompasses acuity, ability to change and sustain focus, and symmetry) and brain processing (integration) of the object seen with the eyes [37].
  - Visual integrity: Includes the visual field (i.e., area of vision where an object can be seen without moving the eyes), the visual acuity (i.e., capacity to distinguish letters and objects clearly) and the presence of eye problems interfering with vision such as strabismus, nystagmus, or problems with fixation of moving objects.
  - Visual perception: The brain’s ability to make sense of what the eyes see, such as visual closure, figure/ground, or visual attention.
  - Visual anticipatory pattern: Ocular movements and gaze time in preparation of upper limb movements.

#### 2.2.2. Descriptions of Upper Limb Functions

Capacities such as joint mobility, strength and motor coordination are required to perform basic daily activities [38]. For the purpose of this review, upper limb functions were separated into two categories:

1. Unilateral motor functions (UMF): This refers to activities performed using only one arm (e.g., reaching, grasping, and releasing).
2. Bilateral motor functions (BMF): This refers to activities performed using both arms simultaneously. Almost all daily living activities can be considered BMF, as they typically require coordination between both arms (e.g., cooking, getting dressed).

### 2.3. Quality Assessment

Two authors (I.P. and O.M.) independently rated the overall quality of each article included in this study, using the “Standard quality assessment criteria” from Kmet et al. [39]. A calibration meeting was initially performed with two articles, to ensure a clear understanding of each criterion and thus standardization and reliability of assessments. A second meeting was performed to discuss the criteria for each included article, until a consensus was reached for a score. In the case of any unresolvable disagreement, a third author (C.M.) performed the assessment to reach consensus.

Each criterion was evaluated on a scale of 2 (2 = meets the criterion, 1 = partially meets the criterion without adding bias to the results, 0 = not mentioned, or added bias to the results). The scores were converted to a percentage and a five-level scale was used to characterize the article’s quality: very high quality = 90% and more (VHQ); high quality = 80 to 89% (HQ); moderate quality = 70 to 79% (MQ); low quality = 60 to 69% (LQ); very low quality = 59% or less (VLQ). A weighted Gwet’s coefficient was calculated for each item of the grid to evaluate the pre-consensus inter-rater agreement. The level of agreement was defined as: poor = <0.0, slight = 0.0 to 0.20, fair = 0.21 to 0.40, moderate = 0.41 to 0.60, substantial = 0.61–0.80, and almost perfect = 0.81 to 1.00 [40].

### 2.4. Data Analysis

Conclusions were separated by type of motor functions (UMF or BMF) and by sensory function evaluated (tactile, proprioception, visual). Due to the heterogeneity of assessments used and the characteristics of the population evaluated in each article, only a descriptive synthesis of the results was performed, rather than full a meta-analysis.

The level of evidence was characterized by integrating four domains of concerns: (1) number of studies and participants (imprecision); (2) methodological quality (risk of bias); (3) similarities in methodological setting and outcomes (indirectness); and (4) direction of results (inconsistency). An adapted scale has been used in accordance with the type of protocol, and has been used to characterize the level of evidence for sensory and motor function as follows [41]:

- Strong evidence: Multiple high quality studies with consistent results.
- Moderate evidence: Multiple studies including at least one high quality study, or multiple moderate quality or low quality studies presenting consistent results.
- Conflicting evidence: Multiple studies providing inconsistent results, regardless of methodological quality.
- Limited evidence: Multiple moderate quality or low quality studies with inconsistent results, or only one high quality study.
- Very limited evidence: Only one low quality or moderate quality study.

## 3. Results

### 3.1. Selection Process and Description of the Studies

**Figure 1:**
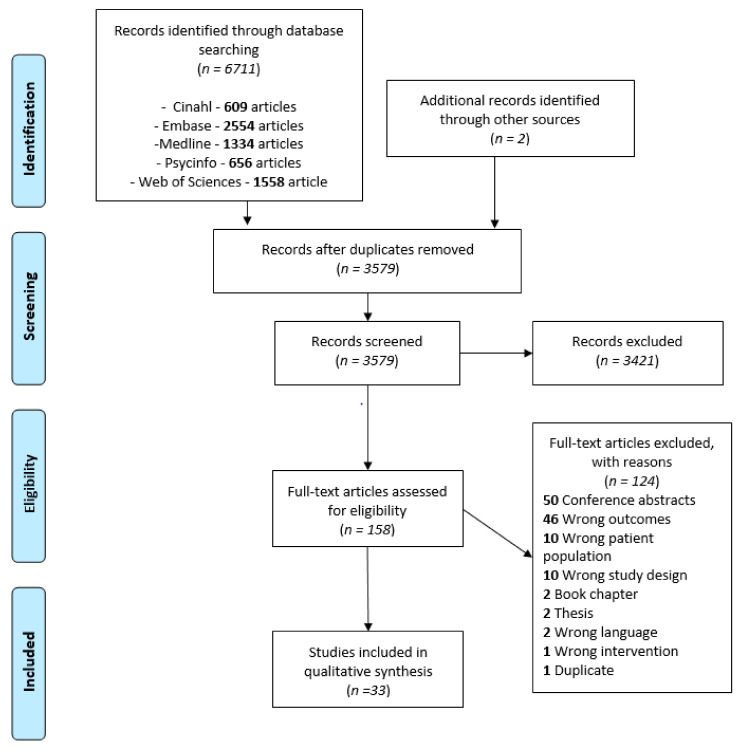
PRISMA flow chart of studies selection process.

The database search strategy yielded 6711 articles, to which two articles were added by manually searching through references. After removing the duplicates, processing titles/abstracts and screening the full texts, 33 articles were included in this review (see Figure 1), representing a total of 1231 participants. Twenty-eight of the articles included children [42,43,44,45,46,47,48,49,50,51,52,53,54,55,56,57,58,59,60,61,62,63,64,65,66,67,68,69], 21 included adolescents [50,52,53,54,55,56,57,58,59,60,62,63,65,66,67,69,70,71,72,73,74] and 4 included adults [62,70,72,74]. Thirty-one of the articles reported results on UMF [42,44,45,46,47,48,49,50,51,52,53,54,55,56,57,58,59,60,62,63,64,65,66,67,68,69,70,71,72,73,74] and 17 on BMF [42,43,44,47,48,55,56,57,59,60,61,63,65,66,67,69,73]. Twenty-five of the articles investigated tactile outcomes [42,43,44,45,49,50,51,52,53,54,55,57,58,59,60,61,62,63,64,65,66,67,70,71,72], 10 investigated proprioceptive outcomes [42,43,49,50,59,64,69,72,73,74] and 7 investigated visual outcomes [46,47,48,56,60,63,68]. Nine studies included a mixed sample of different CP forms [42,43,48,51,54,58,61,62,70] (e.g., hemiplegia, diplegia, athetoid) while others focused specifically on one type. Fifteen articles reported at least 1 classification of incapacity level, 11 of them using the Manual Ability Classification System (MACS) [44,46,56,59,64,66,67,68,70,71,73] and six using the Gross Motor Function Classification System (GMFCS) [42,44,45,56,57,65]). Thirteen articles gathered data on timing/location of lesions [43,45,48,50,55,59,63,67,68,69,71,72,73], but only 1 analyzed [73] the data according to that data and only 2 studies performed statistical analyzes on corticospinal tract lateralization [67,72]. These factors were therefore not taken into account in data synthesis.

### 3.2. Methodological Quality

Kmet and Lee’s Standard quality assessment [39] criteria scores ranged from 54.5% to 95.5% with a mean (±standard deviation) of 78.9 ± 10.9% (see Supplementary Files, Table S3). Six articles were identified as studies of very high quality [43,47,59,65,69,73], 13 as high quality [44,46,50,53,54,56,64,66,67,68,70,71,74], 7 as moderate quality [42,48,49,52,55,57,60], 4 as low quality [45,51,58,72], and 3 as very low quality [61,62,63]. Frequent limitations included failure to report classification of impairment level (46%) or failure to control risk of bias by not considering the heterogeneity of samples (e.g., reporting results without separating them by CP subtype [29%]). The pre-consensus inter-rater agreement of quality assessment was considered as almost perfect (Gwet = 0.969) [40]. When looking at individual criteria the level of agreement was either substantial or almost perfect for 10 out of the 11 items rated (interrater reliability coefficient ranging from 0.63 to 0.97). A moderate level of agreement was found for only one criterion, which was the study design details provided in the article (interrater reliability coefficient of 0.56 (moderate). One potential explanation for this discrepancy could be that the different backgrounds of the two reviewers (occupational therapist and neuropsychologist) may have influenced the details required to earn a perfect score on this specific criterion. However, this potential bias was clarified between the reviewers and a consensus was obtained for each rating.

### 3.3. Association Between Sensory Functions and Motor Functions

**Figure 2:**
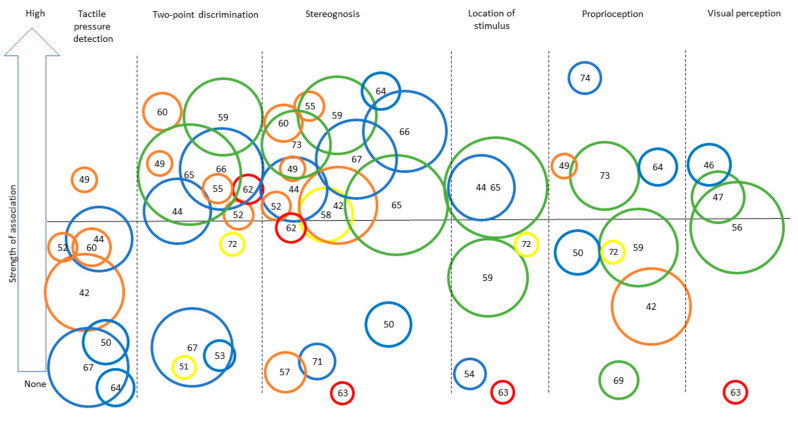
Synthesis of the strength of the association found across studies assessing different sensory outcomes in relation to unilateral motor performance. Legend: Each number represents an article (as identified in the references list). Only categories with more than three articles for UMF are represented to ensure legibility. Level of evidence is represented by the circle color: Green = very high quality study, blue = high quality study, orange = moderate quality study, yellow = low quality study, red = very low quality study. Size of the circle represents the number of participants included in each study (for example, a larger circle means more participants were included) numbers for Figure 2 and Figure 3: [42], Arnould et al. (2014); [43], Arnould et al. (2007); [44], Auld et al. (2012); [46], Bumin et al. (2010); [47], Burtner et al. (2006); [49], Cooper et al. (1995); [50], Duque et al. (2003); [51], Eliasson et al. (1995); [52], Gordon et al. (1999); [53], Gordon et al. (2006); [54], Guedin et al. (2018); [55], Gupta et al. (2017); [56], James et al. 2015; [55], James et al. (2017); [58], Kinnucan et al. (2010); [59], Klingels et al. (2012); [60], Krumlinde-Sundholm et al. (2002); [61], Kurtaran et al. (2015); [62], Law et al. (2008); [63], O’Malley et al. (1977); [64], Robert et al. (2013); [65], Russo et al. (2019); [66], Sakzewski et al. (2010); [67], Simon-Martinez et al. (2018); [69], Woodward et al. (2015); [71], de Campos et al. (2014); [72], Guzzetta et al. (2006); [73], Kuczynski et al. (2016); [74], van Roon et al. (2005).

**Figure 3:**
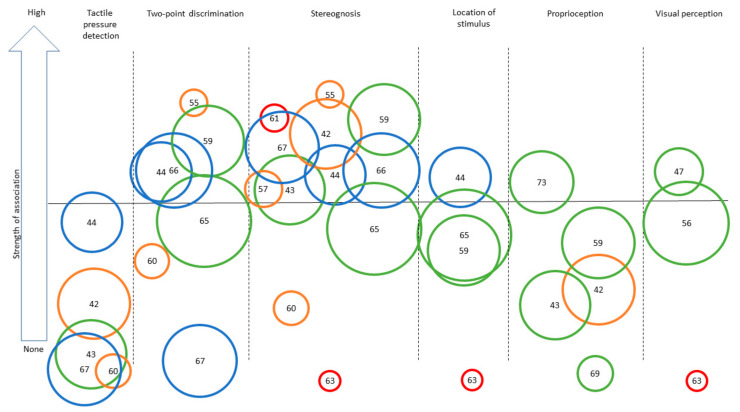
Synthesis of the strength of the association found across studies assessing different sensory outcomes in relation to bilateral motor performance. Legend: Each number represents an article (as identified in the references list). Only categories with more than three articles for BMF are represented to ensure legibility. Level of evidence is represented by the circle color: Green = very high quality study, blue = high quality study, orange = moderate quality study, yellow = low quality study, red = very low quality study. Size of the circle represents the number of participants included in each study (for example, a larger circle means more participants included). The same numbers are used for Figure 2 and Figure 3: [42], Arnould et al. (2014); [43], Arnould et al. (2007); [44], Auld et al. (2012); [46], Bumin et al. (2010); [47], Burtner et al. (2006); [49], Cooper et al. (1995); [50], Duque et al. (2003); [51], Eliasson et al. (1995); [52], Gordon et al. (1999); [53], Gordon et al. (2006); [54], Guedin et al. (2018); [55], Gupta et al. (2017); [56], James et al. 2015; [55], James et al. (2017); [58], Kinnucan et al. (2010); [59], Klingels et al. (2012); [60], Krumlinde-Sundholm et al. (2002); [61], Kurtaran et al. (2015); [62], Law et al. (2008); [63], O’Malley et al. (1977); [64], Robert et al. (2013); [65], Russo et al. (2019); [66], Sakzewski et al. (2010); [67], Simon-Martinez et al. (2018); [69], Woodward et al. (2015); [71], de Campos et al. (2014); [72], Guzzetta et al. (2006); [73], Kuczynski et al. (2016); [74], van Roon et al. (2005).

Table S2 (see Supplementary Files) presents a complete overview of the extracted data. Descriptions of the specific assessment tools used to assess UMF and BMF are presented in Table 1. The assessment tools for UMF are all objective measurements that report quantitative results about the participants’ motor skills. In contrast, the assessment tools of BMF include both subjective assessments (i.e., perception of participants of their motor capacities; *n* = 5) and objective measurements (i.e., quantitative measure of motor function; *n* = 13). The study’s results for both UMF and BMF are presented according to targeted functions in the following order: tactile, proprioceptive, and visual functions. Table 2 presents a synthesis of the results, together with the level of evidence for each result, using the same structure as in the main text. Figure 2 and Figure 3 provide a visual representation of the associations observed for UMF and BMF, respectively.

#### 3.3.1. Tactile Functions

**Tactile pressure detection** was assessed in eight of the articles in relation to UMF [42,44,49,50,52,60,64,67] and in five articles in relation to BMF [42,43,44,60,67]. The results gathered for UMF show conflicting evidence with four articles presenting moderate associations, one showing low significant association, and three showing no to low association. The results are presented for five different UMF assessments (Jebsen-Taylor Test of Hand Function (JTTHF), Melbourne Unilateral Upper Limb Assessment (MUUL), range of motion (ROM), grip strength, and a pick-up task) with inconsistencies across conclusions for the same assessment. For BMF, two articles [42,44] report low and moderate significant association and three articles report no association [43,60,67], meaning that there is a little evidence of association between BMF and tactile pressure detection. Results are presented for three BMF assessments (Assisting Hand Assessment (AHA), ABILHAND-Kids questionnaire and subjective bimanual performance perception) with inconsistent findings.

**Tactile vibration** was assessed in only one high quality article in relation to UMF [70]. As a result, the level of evidence remains limited, but this article does report a high significant association (r > 0.6, *p* < 0.01) for a piano-related task.

**Two-point discrimination** was assessed in 13 of the articles in relation to UMF [44,49,51,52,53,55,59,60,62,65,66,67,72] and in 7 articles in relation to BMF [44,55,59,60,65,66,67]. The results gathered for UMF show conflicting evidence, with nine articles presenting moderate to high associations between two-point discrimination and UMF assessments, three articles reporting no significant association, and one article showing conflicting results (different levels of association depending on the corticospinal tract laterality [72]). Since the majority of high quality studies showed at least a moderate association, the results seem to favor a moderate association between UMF and two-point discrimination. The results are presented for nine different UMF assessments: JTTHF, MUUL, a pick-up test (two different protocols), The South Australian Cerebral Palsy Register classification, grip strength, ROM, and grasping pattern assessments (two different protocols). For BMF, six out of seven articles showed a moderate to high association (r > 0.4, *p* < 0.05) between two-point discrimination and BMF, with only one article [67] presenting an opposite conclusion, meaning that there is moderate evidence of association. Results are presented for three different BMF assessments (AHA, ABILHAND-Kids questionnaire and CP Register Assessment of Bimanual Upper Limb Function), with consistent findings.

**Stereognosis** was assessed in 17 of the articles in relation to UMF [42,44,49,50,52,55,57,58,59,60,62,63,64,65,66,67,71] and in 11 articles in relation to BMF [42,43,44,55,57,59,60,61,65,66,67]. The results gathered show moderate level of evidence for a moderate to high associations between stereognosis and UMF. For UMF, 13 articles reported a significant association (all articles = r > 0.4) while four articles showed no significant effect (these four studies (two high quality, one low quality, and one very low quality represented only 80 participants out of 744)). Results are presented for 11 different UMF assessments: JTTHF, MUUL, ROM, grip strength, a reaching task, The South Australian Cerebral Palsy Register classification, Box and Block, Purdue Pegboard, Beery-Buktenica Developmental Test, a pick-up task, and Shriners Hospitals Upper Extremity Evaluation (SHUEE). For BMF, 10 of the 11 articles reported a moderate to high relationship between stereognosis and BMF, meaning that there is a strong evidence of association between them. Only two articles showed contradictory results, one being very low quality [63] and the second using an assessment that differed from all other studies [60]. Results are presented for four different BMF assessments (AHA, ABILHAND-Kids questionnaire, a functional level assessment and CP Register Assessment of Bimanual Upper Limb Function) with consistent findings.

**Graphesthesia** was assessed in three of the articles in relation to UMF [63,65,72] and in one in relation to BMF [65]. Evidence of association between graphesthesia and either UMF or BMF is limited, respectively due to the inconsistent results available and to the limited number of studies available. A low to moderate association is observed in two articles for UMF and in the only study for BMF. One article pointed out the difference of association strength according to CST laterality [72].

**Directionality of stimulation** was assessed in three of the articles in relation to UMF [45,49,71]. The results are contradictory, with one study showing a moderate association between UMF and directionality of stimulation, one reporting a low association and one showing no association. As a result, no definitive conclusions can be reached for this sensory function. The various protocols used across studies might help to explain the differences observed.

**Location of stimulus** was assessed in six articles for UMF [44,54,59,63,65,72] and in three articles in relation to BMF [44,59,65]. Results gathered show conflicting evidence, with four articles reporting moderate associations between UMF and location of stimulus and two articles reporting no association. The four articles reporting an association are of very high, high, or moderate quality, while the two other articles are of high and very low quality. Results are presented for five UMF assessments (MUUL, JTTHF, the South Australian Cerebral Palsy Register classification system, nine-hole peg test and the Beery-Buktenica Developmental Test) in the six different articles, which might help to explain the heterogeneity of results. One study analyzed data according to corticospinal tract laterality and showed that it contributes to the level of association between motor and sensory function [72]. Another article reported higher heterogeneity for certain CP subtypes [54]. For BMF, three articles reported at least a moderate association with AHA and/or ABILHAND-Kids questionnaire, meaning that there is a moderate evidence of an association between BMF and location of stimulus.

**Double simultaneous stimulation** was assessed in three articles in relation to UMF [43,63,72] and in one article in relation to BMF [44]. Limited evidence of association between either UMF or BMF and double simultaneous stimulation is present due to the limited number of studies included in this review. A moderate association is observed in one of the three articles for UMF and in the only study for BMF.

**Temporal discrimination** was assessed in one high quality article evaluating UMF [71]. This article reports no significant association for different parts of a reaching task. As a result, there is limited evidence of an absence of association between temporal discrimination and UMF.

**Texture discrimination** was assessed in two articles in relation to UMF [44,51] and in one article in relation to BMF [44]. Results are similar for both types of motor functions, with no significant effect found, which suggests an absence of association between motor functions and texture discrimination. However, since there were only two studies addressing this topic, this conclusion is arguably weak.

Overall, studies on tactile functions show evidence of moderate to strong association between both two-point discrimination and stereognosis and UMF and BMF, although the evidence remains somewhat conflicting for the association between two-point discrimination and UMF. Other variables received limited attention, and evidence is therefore either limited or conflicting, making it impossible to reach a clear conclusion within the scope of this study.

#### 3.3.2. Proprioceptive Functions

**Proprioception** was addressed in nine articles with respect to UMF [42,49,50,59,64,69,72,73,74] and in five articles in relation to BMF [42,43,59,69,73]. Evidence of association between proprioception and UMF is conflicting, however, with seven articles showing a low to high level of association, two high quality studies reported no association. Evidence for association between proprioception and BMF is scarce, with five articles reporting moderate to no association. The proprioceptive assessments performed were heterogeneous with three different types of assessment: active, passive, and arm position matching (robotic or clinical assessment) reporting different levels of association, and the (robotic assessment showing higher level of association except for [69]). In addition, four joints were assessed across the different articles (fingers, wrist, elbow, and shoulder).

Overall, there is conflicting evidence about the association between UMF or BMF and proprioceptive functions.

#### 3.3.3. Visual Functions

**Vision integrity** (e.g., good visual acuity, absence of malformation, complete visual field) and **visual anticipatory patterns** were assessed in only one study of low quality [46] and one study of high quality [68], respectively. They both report moderate to high association with a very limited level of evidence.

**Visual perception** was assessed in four articles in relation to UMF [46,47,56,63] and in three articles in relation to BMF [47,56,63]. Results gathered show a moderate to high association between visual perception for both UMF and BMF. Only one low quality study reports an absence of association [61], which leads to a moderate level of evidence for UMF and a limited level of evidence for BMF.

Overall, the results support the presence of a moderate to high association between visual functions assessed and motor functions (either UMF or BMF). However, evidence remains limited due to the small number of high quality studies (especially for vision integrity).

## 4. Discussion

This systematic review allows us to conclude that stereognosis and two-point discrimination are both positively associated with motor functions (either UMF or BMF). Other tactile functions have shown conflicting or very limited evidence of association with either UMF or BMF, reflecting the importance of assessing these variables in future studies to clarify their potential contribution to motor impairments. The same conclusion applies to proprioceptive functions, as the heterogeneity across existing studies included in this review makes it impossible to reach a definitive conclusion. A positive association between visual and motor functions has been observed, but the very limited number of high quality studies restrict the strength of this conclusion, especially for vision integrity. First, we will discuss some disparities between the results obtained for UMF and BMF, as to the best of our knowledge this review is the first attempting to synthetize the evidence on the association between BMF and sensory functions. Second, the results of each type of sensory function will be discussed separately (tactile, proprioceptive, and then visual functions). Third, whether the observed associations reflect causal relationships or not will be addressed. Finally, the study limitations and risk of bias will be discussed.

The level of association between sensory functions and BMF appears to be generally similar to that observed with UMF, although studies were generally not designed to address this question directly. The small disparities observed between UMF and BMF conclusions are likely to be explained in part by the type of motor assessments (UMF was always evaluated with objective assessments while BMF was assessed based on self-report in several studies) and the number of high quality studies included for each motor function (which was lower for BMF) [75]. Another notable difference is that UMF assessments generally targeted a specific motor function (e.g., fine or global motor function), while the BMF assessments generally assessed performance in activities of daily living. This needs to be kept in mind when interpreting the results and makes the question of the relative impact of sensory function on UMF and BMF difficult to address. Nevertheless, it would be possible to compare the assessment of unilateral and bilateral functions that would be more similar in terms of task demands and assessment type (i.e., objective measure of performance).

For tactile functions specifically, a previous review by Bleyenheuft and Gordon [76] provided evidence of association between stereognosis and precision grip strength. This strong association could be explained by the fact that stereognosis tests often allow the participant to use movement to gain information about the tactile stimulus. The present systematic review expands these findings by showing that stereognosis is related to a large variety of upper limb motor tasks. It also shows an association between two-point discrimination and a large array of upper limb tasks, while failing to identify associations with other tactile functions. Inconsistent results were found for tactile pressure detection, and evidence was too limited for other tactile functions. These findings support the idea that tactile perception (i.e., tactile functions requiring integration and interpretation of sensory input) is more closely associated with motor deficits than tactile registration (i.e., detection of stimuli). This is not surprising, given that the perception of somatosensory stimuli has been shown to be more systematically impaired than their registration [26] and that there is an alteration of sensory integration in people living with CP [26,76,77]. However, a minimally preserved tactile registration is a pre-requisite to tactile perception [44]. This could contribute to explaining the variability of results for tactile pressure detection: people with milder deficits might be able to compensate for them but not people with larger deficits, leading to a non-linear relationship with motor functions. Another factor that could account for the general heterogeneity of the results for tactile functions is the fact that the clinimetric properties of the test in children with CP are variable and sometimes limited, as reviewed by Auld and collaborators [35]. The assessment of somatosensory functions requires that the task be both understood and performed, which may limit the use of these tests in children with cognitive impairments [78].

The evidence of an association between proprioceptive and UMF or BMF functions is conflicting, with a limited number of studies addressing BMF. However, the association between proprioception and motor functions [79] has been demonstrated, as have some improvements in motor functions when given a specific proprioceptive training for adult stroke survivors [80], highlighting the potential contribution of proprioception to motor functions in other populations, such as in individuals living with CP. Three different types of proprioceptive assessment were used in the included studies: joint matching position assessments, and detection of active and passive joint displacement. The reliability of these assessments has been questioned in the past few years with the lack of protocol standardization and the validity of measurements being pointed out [81,82,83]. Two of these studies used a robotic system that showed good reliability in individuals living with stroke [84,85]. These two studies report conflicting results, with one showing an association with the MUUL [69] and the AHA and the other finding a moderate association with the Purdue Pegboard [73] and the AHA. This discrepancy could be explained by the fact that the unimanual assessment respectively focused on the quality of movement (MUUL) vs. fine motor functions (Purdue Pegboard), but this cannot account for the difference observed for BMF (AHA being used in both studies). The joint being evaluated might also influence the association with motor functions: the results are not conflicting for the wrist and the thumb (all show a significant association with motor function) [42,43,49,64,73,74] but are variable for the index [49,50,59,72], the elbow [64,69,73,74], and the shoulder [64,69,73,74]. Moreover, single joint assessments (e.g., isolated finger movements) and single plane assessments (e.g., detection of flexion extension motions) could contribute to the weak association with BMF, since ADL requires movements of more than one joint on several planes of movement. One study reported [72] a significant association only when controlling for the lateralization of the corticospinal tract (i.e., ipsilateral vs. contralateral vs. mixed side projections) which suggests that neuroplasticity occurring in this motor tract influences the association between proprioception and motor functions. In addition to this pathway, the corpus callosum [86] is known to be involved in BMF and could also mediate the association observed, which suggests that considering the integrity of the pathway could help us understand the association between motor and sensory functions.

Ultimately, evidence is too scarce for definitive conclusions on the association between visual and motor functions (with a total of only six articles for both UMF and BMF), but is generally supportive of the presence of an association. The importance of vision for motor planning (e.g., exploring the environment or the object to adapt the response [87]), motor control (e.g., online control [88]) and motor learning (e.g., use of visual cue to enhance motor performance [89]) are already well known [90], but the impact of visual deficits on motor performance in people living with CP remains unclear. Further studies are required to evaluate the effect of visual integrity and visual perception on motor performance in a real-world context. This is of particular importance given the fact that some interventions that target motor functions rely heavily on visual functions, for example the increasing use of virtual reality or other serious gaming approaches [91].

Study Limitations: The heterogeneity in population characteristics limits the possibility to generalize results: only a few studies reported the classification of impairment level or presented subgroup analyses according to type of CP or individuals’ age. Identification of the MACS and GMFCS would have made it possible to determine whether the samples of studies included in this review are representative of the population of people living with CP. Furthermore, the use of a mix of participants with various anatomical distribution of CP (e.g., hemiplegia, diplegia) in the same article could have contributed to the heterogeneity of the results. However, these characteristics were taken into consideration during the methodological assessments, leading to a lower methodological score and less weight being given in the analysis process. The wide range of ages covered by the articles (sometimes ranging from young children to adult participants within the same study) weakened the conclusions by neglecting to consider the developmental stage of the participants in their interpretations. Bleyenheuft and al. [76] recommended further studies for normative data of sensory functions by age and studies describing the evolution of sensory functions with ageing in people living with CP.

Risk of Bias: Eighty percent (26 out of 33 studies) of the included articles were rated as moderate to very high quality studies, which suggests a low risk of bias. Two articles were also excluded because they were written in a language other than English or French, which adds a small risk of bias. The Gwet coefficient is high (interrater reliability coefficient = 0.969). For all of these reasons, the risk of bias for this systematic review is considered to be very low.

## 5. Conclusions

This systematic review aimed to provide a comprehensive summary of the current evidence related to associations between sensory and motor functions. The level of association between sensory and motor functions was found to vary across the sensory functions evaluated. Stereognosis and two-point discrimination are the two functions that were quite systematically found to be associated with motor functions. While there are some results supporting the associations with proprioception and visual perception as well, evidence is still lacking and there is still considerable heterogeneity across the studies. Further studies should consider additional variables, such as neurological variables (e.g., lesion location and the reorganization of sensorimotor pathways), type of CP and the potential effects of age. Special attention should also be paid to the type of assessments used to assess motor function, since these often focus on different types of tasks (unimanual or bimanual) or motor capacities (e.g., quality of movement compare to fine motor functions). In addition, since the majority of articles report data for one type of sensory input only, more comprehensive assessments (i.e., evaluation of tactile, proprioceptive and visual functions) would help us understand the relative contribution of each sensory modality to simple and more complex motor functions. Finally, evaluating the impact of sensory functions on the use of each upper limb in the context of everyday activities (for instance quantified through accelerometry) would provide a complementary perspective on the contribution of sensory functions to motor performance.

## Figures and Tables

**Table 1:** Motor functions assessments.

Motor Functions	Assessment Tool	Description	*n*
Unilateral motor function	Jebsen-Taylor Test of Hand Function (JTTHF)	Time taken to perform 6 ADL tasks	8
	Melbourne Unilateral Upper Limb Assessment (MUUL)	Quality of movements assessment based on 16 functional movements	8
	Grip and/or pinch strength (Jamar hydraulic hand dynamometer)	Mean of maximal force exerted across trials	6
	Fine motor function (Purdue Pegboard test or nine-hole peg test)	Mean number of pegs placed within 30 s across 3 trials	4
	Range of motion (ROM)	Complete passive or active range of motion	4
	Gross manual function (Box and blocks test)	Number of blocks carried within 1 min	3
	Grasp pattern (The Functional Evaluation of the Congenitally Anomalous Hand or Functional Hand Grip Test)	Scale from 0 to 8 to rate the grasp pattern	2
	Developmental assessment (Griffith developmental scales)	Hand-eye coordination: based on fine motor skills, manual dexterity and visual perception skill score during play	1
	Motor recovery (Chedoke McMaster Stroke Assessment)	Quality of upper limb motor recovery on a scale of 1 to 7	1
	Piano task	Repeatedly pressing the same key with a single finger	1
	School function assessments (SFA)	Written Work subtest to measure a child’s ability to produce written work	1
	The Beery Developmental Test	Visuomotor integrations, drawing skills	1
	The South Australian Cerebral Palsy Register classification	Ordinal scale identifying the degree of impairment of each upper limb	1
Bilateral motor function	Assisting Hand Assessment (AHA)	Performance score based on bimanual use during play and ADL	9
	ABILHAND-Kids questionnaire	Parent’s perception of child’s capacity to perform ADL	3
	Assessment of Motor and Process Skills (AMPS)	Score of confidence and efficiency on 16 motor tasks and 20 process skills	2
	Developmental assessment (Griffith developmental scales)	Global score (overall development compared to their age-matched peers) and performance score (speed and precision during play)	1
	Pediatric Evaluation of Disability Inventory (PEDI) caregiver	73 capability items in 15 skill areas of task completion	1
	School function assessments (SFA)	Measure a child’s use of classroom tools and the ability to manipulate	1
	Shriners Hospitals Upper Extremity Evaluation (SHUEE)	16 bimanual tasks evaluating tone, spontaneous use of the affected upper limb, passive and active range of motion	1

Legend: *n* = number of studies using this tool; MUUL: Melbourne unilateral upper limb assessment; JTTHF: Jebsen-Taylor Test of Hand Function; School function assessments; ROM: range of motion; AHA: Assisting hand assessment; SHUEE: Shriners Hospitals Upper Extremity Evaluation; AMPS: Assessment of Motor and Process Skills; PEDI: Pediatric Evaluation of Disability Inventory.

**Table 2:** Synthesis of conclusion and level of evidence for each combination of sensory and motor outcomes.

Sensory Outcomes	Type	Motor Outcomes	Number of Studies and Quality	Number of Participants	Results	Conclusion	Overall Quality of Evidence
Tactile	Tactile pressure detection	Unilateral motor function	Total: 8 4HQ 1MQ 3LQ	Total: 344 HQ = 159 (range 16–75) MQ = 25 LQ = 160 (range 9–136)	Significant association: 1HQ showed moderate association with JTTHF and MUUL 1MQ showed moderate association with a pick-up task 3LQ showed moderate association with a JTTHF, ROM and grip strength No significant association: 3HQ with MUUL, JTTHF, grip strength, ROM	Low to moderate association	Conflicting evidence
		Bilateral motor function	Total: 5 1VHQ 2HQ 1MQ 1LQ	Total: 389 VHQ = 101 HQ = 127 (range 52–75) MQ = 25 LQ = 136	Significant association: 1HQ showed moderate association with AHA 1LQ showed low association with subjective performance No significant association: 1VHQ with ABILHAND questionnaire 1HQ with AHA 1MQ with subjective performance in ADL	None to low association	Limited evidence
	Tactile vibration detection	Unilateral motor function	1HQ	Total: 16 HQ = 16	Significant association: 1HQ showed high association with a pick-up task	High association	Limited evidence
		Bilateral motor function	----				
	Two-point discrimination	Unilateral motor function	Total: 13 2VHQ 4HQ 3MQ 3LQ 1VLQ	Total: 533 VHQ = 189 (range 81–108) HQ = 217 (range 20–75) MQ = 49 (range 12–25) LQ = 48 (range 9–24) VLQ = 30	Significant association: 2VHQ and 2HQ showed moderate to high association with The South Australian Cerebral Palsy Register classification, JTTHF and MUUL 1MQ showed high association with a pick-up test 1MQ showed moderate to high association with MUUL, but only when the type of CST wiring was controlled for 3LQ showed moderate to high association with JTTHF and strength 1 VLQ showed moderate association with MUUL No significant association: 2HQ with JTTHF and grip strength 1MQ with grasping task	Moderate to high association	Conflicting evidence
		Bilateral motor function	Total: 7 2VHQ 3HQ 1MQ 1LQ	Total: 435 VHQ = 189 (range 81–108) HQ = 197 (range 52–75) MQ = 25 LQ = 24	Significant association: 2VHQ showed moderate to high association with CP Register Assessment of Bimanual Upper Limb Function and ABILHAND-Kids questionnaire 2HQ showed moderate to high association with AHA 1MQ showed moderate association with a subjective questionnaire of performance in everyday tasks only for the 7 mm discrimination test 1LQ showed high association with AHA No significant association: 1HQ with AHA	Moderate to high association	Moderate evidence
	Stereognosis	Unilateral motor function	Total: 17 3VHQ 6HQ 2MQ 5LQ 2VLQ	Total: 744 VHQ = 189 (range 81–108) HQ = 238 (range 9–75) MQ = 66 (range 41–65) LQ = 221 (range 9–136) VLQ = 48 (range 18–30)	Significant association: 2VHQ showed moderate to high association with MUUL, The South Australian Cerebral Palsy Register classification 4HQ showed moderate to high association with MUUL, JTTHF and grip strength 2MQ showed moderate to high association with JTTHF and a pick-up task 4LQ showed moderate to high association with JTTHF, grip strength, Box and block, Purdue pegboard and a grasping task 1VLQ showed moderate association with MUUL No significant association: 1HQ with a reaching task 1HQ with MUUL 1LQ with ROM, SHUEE and Box and block 1VLQ with Beery Developmental test	Moderate to high association	Moderate evidence
		Bilateral motor function	Total: 12 3VHQ 3HQ 1MQ 3LQ 2VLQ	Total: 763 VHQ = 290 (range 81–108) HQ = 197 (range 52–75) MQ = 25 LQ = 197 (range 24–136) VLQ = 54 (range 18–36)	Significant association: 3VHQ showed moderate to high association with AHA, ABILHAND-Kids questionnaire and CP Register Assessment of Bimanual Upper Limb Function 3HQ showed moderate to high association with AHA 3LQ showed moderate to high association with AHA and ABILHAND-Kids questionnaire 1VLQ showed moderate to high association with functional level No significant association 1MQ with video-taped bimanual performance 1VLQ with Beery Developmental test	Moderate to high association	Strong evidence
	Graphesthesia	Unilateral motor function	Total: 3 1VHQ 1MQ 1VLQ	Total: 138 VHQ = −108 MQ = 12 VLQ = 18	1VHQ showed moderate association with The South Australian Cerebral Palsy Register classification and robotic task 1MQ showed moderate association for IpsiCST wiring group No significant association: 1VLQ with Beery Developmental Test	Low to moderate association	Limited evidence
		Bilateral motor function	Total: 1 1VHQ	Total: 108 VHQ = 108	Significant association: 1VHQ showed low to moderate association with The South Australian Cerebral Palsy Register classification and robotic task	Low to moderate	Limited evidence
	Directionality	Unilateral motor function	Total: 3 1HQ 1MQ 1VLQ	Total: 32 HQ = 11 MQ = 12 VLQ = 9	Mixed results 1HQ showed moderate association with grasping task 1MQ showed no association for the whole sample, except for the left lesion group 1LQ showed association for 6 out of 9 with grasping, range of motion, and grip strength	Moderate association	Conflicting evidence
		Bilateral motor function			----		
	Location of stimulus	Unilateral motor function	Total: 6 2VHQ 2HQ 1MQ 1VLQ	Total: 282 VHQ = 189 (range 81–108) HQ = 63 (range 11–52) MQ = 12 VLQ = 18	Significant association: 2VHQ showed moderate association with MUUL and The South Australian Cerebral Palsy Register classification 1HQ showed moderate association with MUUL and JTTHF No significant association: 1HQ with nine-hole peg test 1MQ showed no association except for the ipsiCST wiring group 1VLQ with the Beery Developmental Test	Moderate association	Conflicting evidence
		Bilateral motor function	Total: 3 2VHQ 1HQ 1VLQ	Total: 259 VHQ = 189 (range 81–108) HQ = 52 VLQ = 18	Significant association: 2VHQ showed moderate association with AHA and ABILHAND-Kids questionnaire 1HQ showed moderate to high association with AHA No significant association 1VLQ with the Beery Developmental Test	Moderate association	Moderate evidence
	Double simultaneous stimulation	Unilateral motor function	Total: 3 1HQ 1MQ 1VLQ	Total: 82 HQ = 52 MQ = 12 VLQ = 18	Significant association: 1HQ showed moderate association with MUUL and JHTTF No significant association: 1MQ with MUUL (different results according to CST laterality) 1VLQ with Beery Developmental Test	Moderate association	Limited evidence
		Bilateral motor function	Total: 1 1HQ	Total: 52 HQ = 52	Significant association: 1HQ showed moderate association with AHA	Low to moderate association	Limited evidence
	Temporal discrimination	Unilateral motor function	Total: 1 1HQ	Total:11 HQ = 11	1HQ showed no to low association for different components of reaching	No to low association	Limited evidence
		Bilateral motor function	----				
	Texture	Unilateral motor function	Total: 2 1HQ 1MQ	Total: 64 HQ = 52 MQ = 12	No significant association: 1HQ showed no association with MUUL and JTTHF 1MQ showed	No association	Limited evidence
		Bilateral motor function	Total: 1 1HQ	Total: 52 HQ = 52	No significant association: 1HQ showed no association with AHA	No association	Limited evidence
Proprioception	Passive or active or joint matching position	Unilateral motor function	Total: 9 3VHQ 3HQ 1MQ 2LQ	Total: 335 VHQ = 181 (range 17–81) HQ = 40 (range 8–16) MQ = 12 LQ = 145 (range 9–136)	Significant association: 3VHQ and 2 HQ showed moderate to high association with MUUL, wrist strength, ROM and a robotic task 1MQ showed moderate association for IpsiCST wiring group with MUUL 2LQ showed low to moderate association with grip strength, ROM and grasping No significant association 1VHQ with MUUL 1HQ with MUUL	Moderate association	Conflicting results
		Bilateral motor function	Total: 5 4VHQ 1LQ	Total: 375 VHQ = 239 (range 17–101) LQ = 136	Significant association: -3VHQ showed low to high association with AHA, a robotic task and ABILHAND-kids questionnaire -1LQ with ABILHAND questionnaire No significant association 1VHQ with AHA	Low to moderate association	Conflicting results
Visual	Visual field/Visual acuity, Vision integrity (fixation, nystagmus, strabismus)	Unilateral motor function	Total: 1 1LQ	Total: 22	Significant association: 1LQ showed moderate association with Griffith developmental scales	Moderate association	Very limited evidence
		Bilateral motor function	Total: 1 1LQ	Total: 22	Significant association: 1LQ showed moderate to high association with Griffith developmental scales	Moderate association	Very limited evidence
	Visual perception	Unilateral motor function	Total: 4 1VHQ 2HQ 1VLQ	Total: 175 VHQ = 20 HQ = 127 (range 26–101) VLQ = 18	Significant association: 1VHQ showed moderate association with written work 2HQ showed moderate to high association with written work and JTTHF No significant association: 1VLQ with Beery Developmental Test	Moderate association	Moderate evidence
		Bilateral motor function	Total: 3 1VHQ 1HQ 1VLQ	Total: 121 VHQ = 20 HQ = 101	Significant association: 1VHQ and 1HQ showed moderate to high association for written work No significant association −1 VLQ with Beery Developmental Test	Moderate to high association	Limited evidence
	Visual anticipatory pattern	Unilateral motor function	Total: 1 1HQ	Total: 13 HQ = 13	Significant association: 1HQ showed association on reaction time and speed of movement when compare to CTRL during a reaching task	Moderate association	Limited evidence
		Bilateral motor function	----				

Legend: VHQ: very high quality study; HQ: high quality; MQ: moderate quality; LQ: low quality; VLQ: very low quality; MUUL: Melbourne Unilateral Upper Limb Assessment; JTTHF: Jebsen-Taylor Test of Hand Function; AHA: Assisting hand assessment; AMPS: Assessment of Motor and Process Skills; 2PD: two-point discrimination; ROM: range of motion; CTRL: control; CST: corticospinal; PVI: periventricular infarction, AIS: arterial ischemic stroke; SP: spastic; ATH: athetoid; DQ: developmental quotient.

## Data Availability

The data presented in this study are available in Supplementary Materials (see above).

## Supplementary Materials

The following are available online at https://www.mdpi.com/article/10.3390/brainsci11060744/s1, Table S1: Search strategies for all databases, Table S2: Extracted data for each study, Table S3: Assessment of study quality by an appraisal quality tool (Kmet &al., 2004)
