# Supplementary material for: Impact of Sensory Deficits on Upper Limb Motor Performance in Individuals with Cerebral Palsy: A Systematic Review
Source: Brain Sci. 2021 Jun 3;11(6):744. doi: 10.3390/brainsci11060744 (PMC8227331; doi:10.3390/brainsci11060744)
Supplement: Supplementary file 1 [file brainsci-11-00744-s001.zip › brainsci-1231764-supplementary.pdf]

Supplementary Material

Table S1: Search strategies for all databases

**Medline (Ovid)**	
**1**	((cerebral adj2 (pals* or paresis or pareses or paralys*)) or little* disease* or (spastic adj2 (diplegi* or hemiplegi* or hemipares* or quadripares* or tetrapares*)) or ((congenital* or perinatal* or peri natal* or prenatal* or pre natal* or postnatal* or post natal* or f?etal or infan* or neonat* or neo nat* or newborn* or new born* or utero or early) adj2 (brain or cerebral) adj2 (injur* or lesion* or trauma* or damag*)) or ((perinatal* or peri natal* or prenatal* or pre natal* or postnatal* or post natal* or f?etal or infan* or neonat* or neo nat* or newborn* or new born* or utero) adj stroke*)).ti,ab,kw.
**2**	Cerebral Palsy/
**3**	or/1-2
**4**	(somatosensory or sensory or sensorially or sensation* or sensibilit* or som?esthetic* or propriocept* or kin?esthe* or stereognos* or astereognos* or gnosi* or two point discrimination* or "2 point discrimination*" or (position adj2 sens*) or touch or tactile or haptic or tactual or vision* or visual* or spatial process* or body image or ((movement* or pattern* or space* or spatial or form* or colo?r*) adj3 (detect* or recogni* or percept* or discriminat* or sens*)) or (passive adj2 movement*)).ti,ab,kw.
**5**	((sense or senses or sensing or percept*) adj4 (disturb* or deficit* or problem* or trouble* or dysfunction* or disorder* or deficien* or defect* or disab* or handicap* or loss* or impair* or alterat* or abnormal* or function* or process* perform* or acuity or evaluat* or assess* or measur* or test* or task* or threshold* or stimulus or stimuli or feedback* or process* or respon* or discriminat* or input*)).ti,ab,kw.
**6**	Feedback, Sensory/ or Proprioception/ or Kinesthesis/ or Touch/ or Touch Perception/ or Stereognosis/ or Sensory Thresholds/ or Somatosensory Disorders/ or Evoked Potentials, Somatosensory/ or Sensation/ or Sensation Disorders/ or Perception/ or Perceptual Disorders/ or "Discrimination (Psychology)"/ or Signal Detection, Psychological/ or Differential Threshold/ or Vision/ or Visual Perception/ or Visual Fields/ or Visual Field Tests/ or Color Perception Tests/ or Color Perception/ or Evoked Potentials, Visual/ or Vision Tests/ or Pattern Recognition, Visual/ or Pattern Recognition, Physiological/ or Spatial Processing/ or Space perception/ or Form perception/ or Body Image/
**7**	or/4-6
**8**	(((bimanual or bi manual or bilateral or bi lateral or unilateral or uni lateral or unimanual or uni manual or upper limb* or upper extremit* or hand or hands or arm or arms or finger* or motor or psychomotor or sensorimotor) adj4 (perform* or skill* or abilit* or capacit* or function* or movement* or process* or control* or evaluat* or assess* or measur* or test* or task* or disturb* or deficit* or problem* or

	trouble* or dysfunction* or disorder* or deficien* or defect* or disab* or handicap* or loss* or impair* or alterat* or abnormal* or outcome* or coordinat* or integrat*)) or (task* adj2 (performance or analysis)) or dissymmet* or asymmet* or reach* or grasp* or dexterity or fine motor or gross motor).ti,ab,kw.
**9**	Upper Extremity/ or Arm/ or Hand/ or Fingers/ or exp Psychomotor Performance/ or Motor Skills Disorders/ or Motor skills/
**10**	or/8-9
**11**	3 and 7 and 10

Embase (Ovid)

**1**	((cerebral adj2 (pals* or paresis or pareses or paralys*)) or little* disease* or (spastic adj2 (diplegi* or hemiplegi* or hemipares* or quadripares* or tetrapares*)) or ((congenital* or perinatal* or peri natal* or prenatal* or pre natal* or postnatal* or post natal* or f?etal or infan* or neonat* or neo nat* or newborn* or new born* or utero or early) adj2 (brain or cerebral) adj2 (injur* or lesion* or trauma* or damag*)) or ((perinatal* or peri natal* or prenatal* or pre natal* or postnatal* or post natal* or f?etal or infan* or neonat* or neo nat* or newborn* or new born* or utero) adj stroke*)).ti,ab,kw.
**2**	Cerebral palsy/
**3**	or/1-2
**4**	(somatosensory or sensory or sensorially or sensation* or sensibilit* or som?esthetic* or propriocept* or kin?esthe* or stereognos* or astereognos* or gnosi* or two point discrimination* or "2 point discrimination*" or (position adj2 sens*) or touch or tactile or haptic or tactual or vision* or visual* or spatial process* or body image or ((movement* or pattern* or space* or spatial or form* or colo?r*) adj3 (detect* or recogni* or percept* or discriminat* or sens*)) or (passive adj2 movement*)).ti,ab,kw.
**5**	((sense or senses or sensing or percept*) adj4 (disturb* or deficit* or problem* or trouble* or dysfunction* or disorder* or deficien* or defect* or disab* or handicap* or loss* or impair* or alterat* or abnormal* or function* or process* or perform* or acuity or evaluat* or assess* or measur* or test* or task* or threshold* or stimulus or stimuli or feedback* or process* or respon* or discriminat* or input*)).ti,ab,kw.
**6**	somatosensory system/ or sensory system/ or sensory evaluation/ or sensory feedback/ or proprioception/ or proprioceptive feedback/ or tactile feedback/ or visual feedback/ or kinesthesia/ or touch/ or tactile discrimination/ or perceptive threshold/ or visual threshold/ or abnormal sensation/ or somatosensory evoked potential/ or sensory evoked potential/ or visual evoked potential/ or sensory dysfunction/ or somatosensory disorder/ or movement perception/ or perceptive discrimination/ or color discrimination/ or spatial discrimination/ or visual discrimination/ or differential threshold/ or sensory system examination/ or perception test/ or sensory perception test/ or visual perception test/ or two-point discrimination test/ or vision/ or color vision defect/ or visual system examination/ or vision test/ or color vision test/ or visual field defect/ or visual field/ or visual impairment/ or body image/ or pattern recognition/ or perception/ or sensation/

**7**	or/4-6
**8**	(((bimanual or bi manual or bilateral or bi lateral or unilateral or uni lateral or unimanual or uni manual or upper limb* or upper extremit* or hand or hands or arm or arms or finger* or motor or psychomotor or sensorimotor) adj4 (perform* or skill* or abilit* or capacit* or function* or movement* or process* or control* or evaluat* or assess* or measur* or test* or task* or disturb* or deficit* or problem* or trouble* or dysfunction* or disorder* or deficien* or defect* or disab* or handicap* or loss* or impair* or alterat* or abnormal* or outcome* or coordinat* or integrat*)) or (task* adj2 (performance or analysis)) or dissymmet* or asymmet* or reach* or grasp* or dexterity or fine motor or gross motor).ti,ab,kw.
**9**	upper limb/ or arm/ or hand/ or finger/ or motor control/ or motor coordination/ or motor performance/ or motor dysfunction/ or task performance/ or psychomotor disorder/ or exp psychomotor performance/ or motor function test/ or dexterity test/ or sensorimotor function/ or sensorimotor integration/ or visuomotor coordination/ or eye hand coordination/ or motor dysfunction assessment/
**10**	Or/8-9
**11**	3 and 7 and 10

PsychInfo (Ovid)

**1**	((cerebral adj2 (pals* or paresis or pareses or paralys*)) or little* disease* or (spastic adj2 (diplegi* or hemiplegi* or hemipares* or quadripares* or tetrapares*)) or ((congenital* or perinatal* or peri natal* or prenatal* or pre natal* or postnatal* or post natal* or f?etal or infan* or neonat* or neo nat* or newborn* or new born* or utero or early) adj2 (brain or cerebral) adj2 (injur* or lesion* or trauma* or damag*)) or ((perinatal* or peri natal* or prenatal* or pre natal* or postnatal* or post natal* or f?etal or infan* or neonat* or neo nat* or newborn* or new born* or utero) adj stroke*)).ti,ab,id.
**2**	Cerebral palsy/
**3**	or/1-2
**4**	(somatosensory or sensory or sensorially or sensation* or sensibilit* or som?esthetic* or propriocept* or kin?esthe* or stereognos* or astereognos* or gnosi* or two point discrimination* or "2 point discrimination*" or (position adj2 sens*) or touch or tactile or haptic or tactual or vision* or visual* or spatial process* or body image or ((movement* or pattern* or space* or spatial or form* or colo?r*) adj3 (detect* or recogni* or percept* or discriminat* or sens*)) or (passive adj2 movement*)).ti,ab,id.
**5**	((sense or senses or sensing or percept*) adj4 (disturb* or deficit* or problem* or trouble* or dysfunction* or disorder* or deficien* or defect* or disab* or handicap* or loss* or impair* or alterat* or abnormal* or function* or process* or perform* or acuity or evaluat*

	or assess* or measur* or test* or task* or threshold* or stimulus or stimuli or feedback* or process* or respon* or discriminat* or input*)).ti,ab,id.
**6**	Sensory Feedback/ or Sensory Integration/ or Sensory Integration Dysfunction/ or Proprioception/ or Somatosensory Disorders/ or Sensory System Disorders/ or Perceptual Disturbances/ or Somatosensory Evoked Potentials/ or Somesthetic Perception/ or Kinesthetic perception/ or Tactual Perception/ or "Signal Detection (Perception)"/ or Visual perception/ or Vision/ or Color perception/ or Spatial Perception/ or Visual Feedback/ or Visual Field/ or Form and Shape Perception/ or Body image/ or Body Awareness/ or Perception/ or Pattern Discrimination/ or Perceptual Discrimination/ or Visual Discrimination/ or Visual Thresholds/ or Visuospatial Ability/ or Visual Evoked Potentials/
**7**	or/4-6
**8**	(((bimanual or bi manual or bilateral or bi lateral or unilateral or uni lateral or unimanual or uni manual or upper limb* or upper extremit* or hand or hands or arm or arms or finger* or motor or psychomotor or sensorimotor) adj4 (perform* or skill* or abilit* or capacit* or function* or movement* or process* or control* or evaluat* or assess* or measur* or test* or task* or disturb* or deficit* or problem* or trouble* or dysfunction* or disorder* or deficien* or defect* or disab* or handicap* or loss* or impair* or alterat* or abnormal* or outcome* or coordinat* or integrat*)) or (task* adj2 (performance or analysis)) or dissymmet* or asymmet* or reach* or grasp* or dexterity or fine motor or gross motor).ti,ab,id.
**9**	"Arm (Anatomy)"/ or "Hand (Anatomy)"/ or "Fingers (Anatomy)"/ or Motor Coordination/ or Motor Skills/ or Task analysis/ or Motor Performance/ or Motor Control/ or Perceptual Motor Processes/ or Perceptual Motor Coordination/ or Visual Motor Integration/ or Physical dexterity/ or Sensorimotor Measures/ or Motor Processes/ or Grasping/
**10**	Or/8-9
**11**	3 and 7 and 10
**Cinahl (EBSCO)**	
**1**	TI ((cerebral N1 (pals* or paresis or pareses or paralys*)) or "little* disease*" or (spastic N1 (diplegi* or hemiplegi* or hemipares* or quadripares* or tetrapares*)) or ((congenital* or perinatal* or "peri natal*" or prenatal* or "pre natal*" or postnatal* or "post natal*" or f#etal or infan* or neonat* or "neo nat*" or newborn* or "new born*" or utero or early) N1 (brain or cerebral) N1 (injur* or lesion* or trauma* or damag*)) or ((perinatal* or "peri natal*" or prenatal* or "pre natal*" or postnatal* or "post natal*" or f#etal or infan* or neonat* or "neo nat*" or newborn* or "new born*" or utero) W0 stroke*)) OR<br>AB ((cerebral N1 (pals* or paresis or pareses or paralys*)) or "little* disease*" or (spastic N1 (diplegi* or hemiplegi* or hemipares* or quadripares* or tetrapares*)) or ((congenital* or perinatal* or "peri natal*" or prenatal* or "pre natal*" or postnatal* or "post natal*" or

	f#etal or infan* or neonat* or "neo nat*" or newborn* or "new born*" or utero or early) N1 (brain or cerebral) N1 (injur* or lesion* or trauma* or damag*)) or ((perinatal* or "peri natal*" or prenatal* or "pre natal*" or postnatal* or "post natal*" or f#etal or infan* or neonat* or "neo nat*" or newborn* or "new born*" or utero) W0 stroke*))
**2**	MH (Cerebral palsy)
**3**	S1 OR S2
**4**	TI (somatosensory or sensory or sensorially or sensation* or sensibilit* or som#esthetic* or propriocept* or kin#esthe* or stereognos* or astereognos* or gnosi* or "two point discrimination*" or "2 point discrimination*" or (position N1 sens*) or touch or tactile or haptic or tactual or vision* or visual* or "spatial process*" or "body image" or ((movement* or pattern* or space* or spatial or form* or colo#r*) N2 (detect* or recogni* or percept* or discriminat* or sens*)) or (passive N1 movement*)) OR AB (somatosensory or sensory or sensorially or sensation* or sensibilit* or som#esthetic* or propriocept* or kin#esthe* or stereognos* or astereognos* or gnosi* or "two point discrimination*" or "2 point discrimination*" or (position N1 sens*) or touch or tactile or haptic or tactual or vision* or visual* or "spatial process*" or "body image" or ((movement* or pattern* or space* or spatial or form* or colo#r*) N2 (detect* or recogni* or percept* or discriminat* or sens*)) or (passive N1 movement*))
**5**	TI ((sense or senses or sensing or percept*) N3 (disturb* or deficit* or problem* or trouble* or dysfunction* or disorder* or deficien* or defect* or disab* or handicap* or loss* or impair* or alterat* or abnormal* or function* or process* or perform* or acuity or evaluat* or assess* or measur* or test* or task* or threshold* or stimulus or stimuli or feedback* or process* or respon* or discriminat* or input*)) OR AB ((sense or senses or sensing or percept*) N3 (disturb* or deficit* or problem* or trouble* or dysfunction* or disorder* or deficien* or defect* or disab* or handicap* or loss* or impair* or alterat* or abnormal* or function* or process* or perform* or acuity or evaluat* or assess* or measur* or test* or task* or threshold* or stimulus or stimuli or feedback* or process* or respon* or discriminat* or input*))
**6**	MH (Proprioception or Kinesthesis or Touch or Sensation or Sensation Disorders or Somatosensory Disorders or Perceptual Distortion or Perception or Perceptual Disorders or Evoked Potentials, Somatosensory or Visual Perception or Vision or Vision tests or Evoked Potentials, Visual or Visual Fields or Color Perception or Color Perception Tests or Body Image or Spatial Perception)
**7**	S4 OR S5 OR S6
**8**	TI (((bimanual or "bi manual" or bilateral or "bi lateral" or unilateral or "uni lateral" or unimanual or "uni manual" or "upper limb*" or "upper extremit*" or hand or hands or arm or arms or finger* or motor or psychomotor or sensorimotor) N3 (perform* or skill* or abilit* or capacit* or function* or movement* or process* or control* or evaluat* or assess* or measur* or test* or task* or disturb* or deficit* or problem* or trouble* or dysfunction* or disorder* or deficien* or defect* or disab* or handicap* or loss* or impair* or

	alterat* or abnormal* or outcome* or coordinat* or integrat*)) or (task* N1 (performance or analysis)) or dissymmet* or asymmet* or reach* or grasp* or dexterity or fine motor or gross motor) OR AB (((bimanual or "bi manual" or bilateral or "bi lateral" or unilateral or "uni lateral" or unimanual or "uni manual" or "upper limb*" or "upper extremit*" or hand or hands or arm or arms or finger* or motor or psychomotor or sensorimotor) N3 (perform* or skill* or abilit* or capacit* or function* or movement* or process* or control* or evaluat* or assess* or measur* or test* or task* or disturb* or deficit* or problem* or trouble* or dysfunction* or disorder* or deficien* or defect* or disab* or handicap* or loss* or impair* or alterat* or abnormal* or outcome* or coordinat* or integrat*)) or (task* N1 (performance or analysis)) or dissymmet* or asymmet* or reach* or grasp* or dexterity or fine motor or gross motor)
**9**	MH (Upper Extremity or Arm or Hand or Fingers or Psychomotor Performance or Motor Skills Disorders or Motor Skills or Object Manipulation or "Task Performance and analysis" or Sensory Motor Integration or Reaching)
**10**	S8 OR S9
**11**	S3 AND S7 AND S10
**Web of Science**	
**1**	TS=((cerebral NEAR/1 (pals* or paresis or pareses or paralys*)) or "little* disease*" or (spastic NEAR/1 (diplegi* or hemiplegi* or hemipares* or quadripares* or tetrapares*)) or ((congenital* or perinatal* or "peri natal*" or prenatal* or "pre natal*" or postnatal* or "post natal*" or f$etal or infan* or neonat* or "neo nat*" or newborn* or "new born*" or utero or early) NEAR/1 (brain or cerebral) NEAR/1 (injur* or lesion* or trauma* or damag*)) or ((perinatal* or "peri natal*" or prenatal* or "pre natal*" or postnatal* or "post natal*" or f$etal or infan* or neonat* or "neo nat*" or newborn* or "new born*" or utero) NEAR/0 stroke*))
**2**	TS=(somatosensory or sensory or sensorially or sensation* or sensibilit* or som$esthetic* or propriocept* or kin$esthe* or stereognos* or astereognos* or gnosi* or "two point discrimination*" or "2 point discrimination*" or (position NEAR/1 sens*) or touch or tactile or haptic or tactual or vision* or visual* or "spatial process*" or "body image" or ((movement* or pattern* or space* or spatial or form* or colo$r*) NEAR/2 (detect* or recogni* or percept* or discriminat* or sens*)) or (passive NEAR/1 movement*))
**3**	TS=((sense or senses or sensing or percept*) NEAR/3 (disturb* or deficit* or problem* or trouble* or dysfunction* or disorder* or deficien* or defect* or disab* or handicap* or loss* or impair* or alterat* or abnormal* or function* or process* or perform* or acuity or evaluat* or assess* or measur* or test* or task* or threshold* or stimulus or stimuli or feedback* or process* or respon* or discriminat* or input*))
**4**	#2 OR #3
**5**	TS=(((bimanual or "bi manual" or bilateral or "bi lateral" or unilateral or "uni lateral" or unimanual or "uni manual" or "upper limb*" or "upper extremit*" or hand or hands or arm or arms or finger* or motor or psychomotor or sensorimotor) NEAR/3 (perform* or

	skill* or abilit* or capacit* or function* or movement* or process* or control* or evaluat* or assess* or measur* or test* or task* or disturb* or deficit* or problem* or trouble* or dysfunction* or disorder* or deficien* or defect* or disab* or handicap* or loss* or impair* or alterat* or abnormal* or outcome* or coordinat* or integrat*)) or (task* NEAR/1 (performance or analysis)) or dissymmet* or asymmet* or reach* or grasp* or dexterity or fine motor or gross motor)
**6**	#1 AND #4 AND #5

Table S2: Extracted data for each study.

Authors (years)	Participant characteristics: Age/Sex<br><br>Lesion type and/or timing of lesion	MACS/GMFCS level<br><br>CP type	Motor assessments / outcomes (name of assessment used)	Motor function (UMF or BMF)	Sensory assessments / outcomes (name of the assessment used)	Sensory modality	Results
Alves-Pinto & al. (2017)	CP subjects :<br><br>Adolescents (n=9) (CPY):<br>Age: Mean=15y; Range= 11-17y<br>Sex:<br>4M; 5W<br><br>Adults (n=7) (CPA)<br>Age<br>Mean=44 y, Range= 34-52 y<br>Sex: 3M; 4W<br><br>Lesion type and/or timing: N/R<br><br>Control subjects:<br><br>Typically developing adolescents (n=6):<br>Age: Mean= 12y, Range= 7-17y<br>Sex: 3M; 3W	CPY: MACS:<br>Level I = 2<br>Level II=3<br>Level III=4<br><br>CPA: MACS Level II=4 Level III=1<br>Level IV=1<br>Level V=1<br><br>CPY: Bilateral spastic= 3; Right spastic hemiplegia=2, Dyskinetic=1, Ataxic=2<br><br>CPA: Bilateral spastic=5, Left spastic hemiplegia=1, Ataxic=1	Repetitive keystrokes / Regularity of keystroke	UMF	Perception of vibration on fingers / Proportion of correct responses for each hand	Tactile	Best hand: r = -0.6, p= 0.01<br><br>Other hand: r= -0.6, p=0.006

Arnould & al. (2007)	Children (n=101)<br>Age: Mean= 10y; Range 6-15y<br>Sex: 59M, 42W<br><br>Lesion type and/or timing: 91% perinatal injury	MACS/GMFCS: N/R<br><br>Quadriplegia= 31<br>Diplegia =20<br>Hemiplegia= 50<br>(Left 25; Right=25)	Subjective manual performance / ABILHAND-Kids questionnaire	BMF (subjective)	a. Stereognosis / Number of objects identified on 10 (Manual Form perception test)<br>b. Passive proprioception of thumb and index MCP<br>c. Tactile pressure detection / Force required to bend the thinnest monofilament detected by child (Semmes–Weinstein monofilaments)	Tactile and Proprioception	a. Stereognosis was significantly associated with manual performance for both hand (r=0.48- 0.49 p < 0.001)<br><br>b. Proprioception was significantly associated with manual performance of the non dominant hand but not with the dominant hand (DH= 0.15, p=0.129; NDH= 0.26, p=0.010)<br><br>c. Tactile pressure detection was not significantly associated with manual performance (r= 0.11-0.191, p > 0.1)<br><br>No sensory function contributes significantly to MA (regression model)

Arnould & al. (2014)	Children (n=136)<br>Age: Mean= 10 +/- 2.6y; Range= 6-16y<br>Sex: 80M, 56W<br><br>Lesion type and/or timing: N/R	GMFCS:<br>Level I= 61<br>Level II= 38<br>Level III= 12<br>Level IV= 21<br>Level V= 4<br>Quadriplegia: 35<br>Diplegia: 24<br>Hemiplegia: 77<br>(Left=39; Right=38)	1- Grip strength / Jamar hydraulic hand dynamometer<br>2- Gross manual function/ Box and blocks test<br>3- Fine motor function / Purdue Pegboard test<br>4- Subjective manual performance /ABILHAND-Kids questionnaire	UMF and BMF	a. Stereognosis / Number of objects identified out of 10 (manual Form perception test)<br>b. Passive proprioception / Movement detection of thumb and index MCP<br>c. Tactile pressure detection / Force required to bend the thinnest monofilament detected by child (Semmes–Weinstein monofilaments)	Tactile and Proprioception	a. Stereognosis was significantly associated with the 4 motor tests for each hand (all p-values < 0.01, r between 0.45-0.61).<br>b. Proprioception was significantly associated with the 4 motor tests for each hand (all p-values < 0.01, r between 0.24-.36), except for the grip strength of the dominant hand.<br>c. Tactile pressure detection was associated with gross motor function and fine motor function for both hands (all p-values < 0.001, r between 0.28-.37), and with grip strength and MP only for the nondominant hand (all p-values < 0.05, r between 0.21-.30).
Auld & al. (2012)	Children (n=52)<br>Age: Mean= 12y; Range 8-17y<br>Sex: 29M; 23W<br><br>Lesion type and/or timing: N/R	GMFCS:<br>Level I=34<br>Level II=1 8<br>MACS:<br>Level I=36<br>Level II= 16<br>Hemiplegia: 52 (Left = 23; Right = 29)	1- Quality of movement/ Melbourne Unilateral Upper Limb Assessment [MUUL]<br>2- Time taken to perform 6 ADL tasks / Jebsen-Taylor Test of Hand Function [JTTHF]<br>3- Bimanual used during play and ADL/ Assisting Hand Assessment [AHA]	UMF and BMF	a. Tactile pressure detection / Force required to bend the thinnest monofilament detected by child (filaments of the Semmes–Weinstein)<br>b. Spatial tactile perception: single point localization / Number of monofilament contacts detected on fingers within 12 trials (filaments	Tactile	a. Tactile pressure detection was significantly associated with MUUL, JTTHF and AHA (R^2=0.16-0.25, $p < 0.005$)<br>b. Single tactile perception was significantly associated with MUUL, JTTHF and AHA (R^2=0.28-0.35, $p < 0.005$)<br>c. Two-point discrimination was significantly associated

					of the Semmes–Weinstein)<br>c. Spatial tactile perception: two-point discrimination / Smallest distance in mm between two pressure points detected (DiskCriminator)<br>d. Spatial tactile perception: Double simultaneous / number of monofilament contacts detected on fingers within 24 trials (monofilaments- bristles)<br>e. Stereognosis / Number of objects identified on 9<br>f. Texture tactile perception / Converted score on the tactile discrimination index representing the moment where the child discriminate smoothness from rudeness on a board (Perspex board)		with MUUL, JTTHF and AHA (static: R^2=0.21-0.26, $p < 0.005$, moving: R^2=0.23-0.29, $p < 0.005$)<br>d. Double simultaneous perception was significantly associated with MUUL, JTTHF and AHA (R^2=0.18-0.35, $p < 0.005$)<br>e. Stereognosis was significantly associated with MUUL, JTTHF and AHA (R^2=0.25-0.36, $p < 0.005$)<br>f. Texture was significantly associated with MUUL, JTTHF and AHA (R^2=-0.24-0.2, $p > 0.005$)<br>All significant except for texture
Bleyenheuft & al. (2011)	Children (n=12)<br>Age:<br>Mean=12.5 +/- 2.1y; range 10-16y<br>Sex: 10M; 2W<br>Lesion type and/or timing: Varied	GMFCS:<br>Level I= 8<br>Level II= 4<br>Hemiplegia: 12 (Left = 8; Right = 4)	Fine motor function / Mean number of pegs placed within 30 seconds across 3 trials (Purdue Pegboard test)	UMF	Tactile spatial resolution threshold / Detecting orientation of the tactile stimulus for 10 trials (Grating Orientation Task)	Tactile	1- Whole sample: no association found for the NDH and DH (r = 0.128, p=0.691 and r = 0.322, p=0.307).<br>2- Right lesion group: no association found for both hands (NDH: r=-0.164, p=0.699; DH: r=0.659, p=0.076).<br>3- For the left lesion group, no significant association was found for the NDH (r=0.8, p= 0.2), but there was a significant

							association for the DH (r = -0.96; p<0.01)<br>a. Overlapping figure was associated with legibility and spacing (r= 0.214-0.524, p < 0.05) but not with rate, form, alignment or size (r=0.172-0.348, p > 0.05)<br><br>b. Copying geometric figure was associated with legibility, alignment and spacing (r= 0.515-0.723, p < 0.05) but not with rate, form or size (r=0.092-0.326, p > 0.05)<br><br>c. Overlapping figure was associated with legibility alignment, size and spacing (r= 0.413-0.592, p < 0.05) but not with rate or form, (r=0.138-0.267, p > 0.05)
Bumin & al. (2010)	<u>CP subjects :</u><br><br>Children (n=26)<br>Age: Mean= 115.02 +/- 15.09 months<br>Sex: 15M, 11W<br><br>Lesion type and/or timing: N/R<br><br><u>Control subjects:</u><br><br>Typically developing children (n=32)<br>Age:<br>Mean= 107.42 +/- 14.36 months<br>Sex: 16M; 16W	MACS:<br>Level I=9<br>Level II=12<br>Level III= 5<br><br>Hemiplegia: 26 (left=26)	Handwriting performance / Rate, legibility, space, alignment, size and form	UMF	a. Overlapping figures / Names the overlapping figures on the card<br>b. Copying geometric forms / Copy five geometric forms<br>c. Reproduction of a 2-dimensional model / Reproduce the pattern along side the design	Visual	
Burtner & al. (2006)	<u>CP subjects:</u><br><br>Children (n=20)<br>Age: Mean: 82.4 +/- 22.4 months<br>Sex: Ratio of 2M for 1W<br><br>Lesion type and/or timing: N/R	MACS/GMFCS: N/R<br><br>Hemiplegia: 20 (Left=11; Right=9)	School Function Assessment / 1- Using materials; 2- Written work sections (give a score on a level of function at school)	UMF and BMF	a. Developmental Test of Visual Perception 2nd ed / Perceptual and visuo-motor assessments divide by:<br>- General visual perception quotient [GVPQ]<br>- Motor reduced perception quotient [MRPQ]<br>- Visual motor integration [VMI]	Visual	Regression coefficient:<br><br>a. General visual perception quotient was associated with using materials and written work sections (regression coefficient= 0.57-0.62, p<0.001)<br>b. Motor reduced perception quotient was associated was not associated with using

	<u>Control subjects:</u><br><br>Typically developing children (n=37)<br>Age: Mean 90.6 +/- 24.8 months				b. Motor-Free Visual Perceptual Test Revised / 40 items measuring visual processing without motor involvement (MVPT-R)		materials and written work sections (regression coefficient= 0.57-0.3-0.41, p > 0.01)<br>c. Visual motor integration was associated with using materials (regression coefficient = 0.68, p < 0.001) but not with written work section (regression coefficient = 0.53, p > 0.001)<br>d. Motor-Free Visual Perceptual Test Revised was not associated with using materials and written works sections (regression coefficient=0.34-0.35, p > 0.001)
Cioni &al. (2000)	Children (n=22) Age: Mean: 13 +/- 2.1; range 8-16 months<br><br>Lesion type and/or timing: 9 infants had grade 1 PVL, 6<br>had grade 2, and seven grade 3	MACS/GMFCS: N/R<br><br>Hemiplegia:2; Diplegia: 15; Tetraplegia: 5	Developmental assessment / Developmental quotient (DQ) 1. Global; 2. Hand-eye coordination; 3. Performance (Griffith developmental scales)	UMF and BMF	Sum of all deficits on 5 where abnormality at one test =1 =<br>- Grating acuity / Threshold of acuity of the finest strips width which the subject consistently responds correctly (Teller acuity card)<br>- Visual field / Eye and head movements (kinetic perimetry)<br>- Horizontal optokinetic nystagmus / Number of saccades elicited<br>- Oculomotor behaviour / Abnormality of eyes contact, nystagmus, fixation and	Visual	Kendall coefficient: global-DQ-visual score= -0.659, p<0.01; Hand-DQ-visual score= -0.597, p<0.01; Performance-DQ-visual score= -0.648, p<0.01

					convergence movement (following and fixation)<br>- Strabismus / Eyes alignment		
Cooper & al. (1995)	CP subjects:<br><br>Children (n=9) Age: Mean= 11.25 +/- 5.36y; range 4.33-18.08y<br>Sex: 7M;2W<br><br>Lesion type and/or timing: N/R<br><br>Control subjects:<br><br>Typically developing children (n=41) Age: Mean: 9.88 +/- 3.02y; range 4.42-16y<br>Sex: 20M;21W	MACS/GMFCS: N/R<br><br>Hemiplegia: 9	1-Range of motion / Complete passive range of motion of the elbow, forearm, wrist, thumb, index and little finger<br>2- Muscle tone / Einstein Neonatal Neurobehavioral Assessment Scale<br>3-Grasp pattern / The Functional Evaluation of the Congenitally Anomalous Hand<br>4- Grip and pinch strength / Jamar dynamometer	UMF	a. Tactile pressure detection / Normal pressure detection set at 2.83, if higher categorize as abnormal (filaments of the Semmes–Weinstein),<br>b. Two-point discrimination / Smallest distance in mm between two pressure points detected (DiskCriminator))<br>c. Stereognosis / Number of shapes identified on 10<br>d. Passive proprioception / Detection of the direction of movements at MCP level (thumb, index and little finger)<br>e. Directionality / Identification of the direction of the filament movements along the finger (filaments of the Semmes–Weinstein)	Tactile and Proprioception	Sensory function correlates with motor function in 6 of 9 CP children while the 3 other subjects show no correlation.
De Campos & al. (2014)	CP subjects:<br><br>Adolescents (n=11) Age: Mean=15 +/- 4 years	MACS:<br>Level I=2<br>Level II=4<br>Level III= 1	Reach to lift task one hand at the time / Reach time, hold time, hand orientation error, intralimb coordination	UMF	a. Tactile spatial discrimination / Identify the orientation of domes placed on the hand (Johnson, Van Boven, Phillips (JVP) domes)	Tactile	No significant association was found between any of the motor and sensory outcomes assessed.

	Sex: 5M;2W (not all data reported)<br><br>7 left middle cerebral artery strokes<br><br><u>Control subjects:</u><br><br>Typically developing adolescents and young adults (n=9) Age: Mean= 17 +/- 5y<br>Sex: 1M; 8W	(not all data reported)<br>Hemiplegia with dystonia: 11 (Left= 11)			b. Temporal discrimination / Threshold of electrical stimulation detection<br>c. Stereognosis / Number of objects identified on 8		
Duque & al. (2003)	<u>CP subjects:</u><br><br>Children and adolescents (n=16) Age: Mean= 12.6 +/- 3.4y<br>Sex: 8M;8W<br><br>Lesion type and/or timing: Six patients had extensive hemispheric lesions (group 1), three patients had focal cortical lesions (group 2) and five had subcortical lesions (group 3). One patient had a focal internal capsule lesion (group 4).	MACS/GMFCS: N/R<br><br>Hemiplegia: 16 (Left=5; Right=11)	Quality of movements / Melbourne assessment of unilateral upper limb [MUUL]	UMF	a. Tactile pressure detection / Force required to bend the thinnest monofilament detected by child (monofilaments of the Semmes-Weinstein)<br>b. Stereognosis / Total of objects identified on 10<br>c. Proprioception / Movement detection of thumb and index MCP	Tactile and Proprioception	MUUL was associated with the proprio-thumb (r= 0.59, p=0.025) but not with the tactile pressure detection (r= -0.11, p=0.721), the stereognosis (r= 0.43, p=0.121) and the proprioception index (r= 0.21, p= 0.463)

	Control subjects:<br><br>Typically developing adolescents and children (n=16) Age: Mean 12.8 +/- 3.2y<br>Sex: 8M;8W						
Eliasson & al. (1995)	CP subjects:<br><br>Children (n=12) Age: Range: 6-18y<br><br>Lesion type and/or timing: N/R<br><br>Control subjects:<br><br>Typically developing children (n= 12)<br>Age-matched to CP participants	MACS/GMFCS: N/R<br><br>Hemiplegia: 6; Diplegia:6	Lift a block with their thumb and index / Grip force and load force	UMF	a. Type of texture on the lifting apparatus/ Silk or rough<br>b. Two-point discrimination / Smallest distance detected	Tactile	No relation between the tactile adaptation and the two-point discrimination. CP children did not change their grip force across different textures, unlike control.
Gordon & al. (2006)	Children (n=12) Age: Mean=6.7y; range 4-8y<br>Sex: 6M;6W<br>Adolescents (n=8); Age: Mean: 11.4y; range 9-13y<br>Sex : 5M; 3W<br><br>Lesion type and/or timing: N/R	MACS/GMFCS: N/R<br><br>Hemiplegia: 20<br><br>Children: Left=8, Right=4<br><br>Adolescents:<br>Left=1<br>Right=7	Time taken to perform 6 ADL tasks/ Jebsen-Taylor Test of Hand Function [JTTHF]	UMF	Two-point discrimination / Smallest distance in mm between two pressure points detected	Tactile	JTTHF was not associated with Two-point discrimination for both group (ChildrenCP: r= 0.341, p=0.369; AdolescentsCP: r= 0.542, p=0.165

Gordon & al. (1999)	Children and adolescents (n=15) Age: range 8-14y<br><br>Lesion type and/or timing: N/R	MACS/GMFCS: N/R<br><br>Hemiplegia: 15	1- Pinch strength /Jamar hydraulic hand dynamometer<br>2- Time taken to perform 6 ADL tasks / Jebsen-Taylor Test of Hand Function [JTTHF]	UMF	a. Two-point discrimination / Smallest distance in mm between two pressure points detected (DiskCriminator)<br>b. Tactile pressure detection / Force required to bend the thinnest monofilament detected by child (Semmes-Weinstein monofilaments)<br>c. Stereognosis / Number of objects identified on 8	Tactile	a. Two-point discrimination was associated with pinch (r= -0.61) et JTTHF (r=0.31)<br>b. Tactile pressure detection was associated with pinch (r= -0.19) and JTTHF (r=0.47)<br>c. Stereognosis was associated with pinch (r=0.59) and JTTHF (r= -0.42)<br><br>All significant
Guedin & al. (2018)	<u>CP subjects:</u><br><br>Children and adolescents (n=11)<br>Hemiplegia: Age: Mean: 11.2 +/- 2.3y; Diplegia: 11.1 +/- 2.5y<br>Sex:<br>Hemiplegia: 5M;6W<br>Diplegia: 6M;5W<br><br>Lesion type and/or timing: N/R<br><br><u>Control subjects:</u><br><br>Typically developing children and adolescents:<br>Age: Mean= 11.2 +/- 2.3y<br>Sex: 9M;13W	MACS/GMFCS: N/R<br><br>Hemiplegia (HCP): 11 (Left=6, Right= 5)<br>Diplegia (DCP): 11	Fine motor function / Nine-hole peg test)	UMF	Finger sense / Number of fingers correctly identified when touch bye the evaluator (gnosia)	Tactile	HCP have sensory deficits in their paretic hands and DCP in their both hands. Otherwise, HCP have motor deficits in their both hands as DCP have impairments in their dominant hand compare to CTRL.

Gupta & al. (2017)	Children and adolescents (n=24): Age: Mean: 10.5 +/- 3.3 y<br>Sex: 13M; 11W<br><br>Lesion type and/or timing: 12 periventricular; 7 middle cerebral arteries; 5 not reported or other	MACS/GMFCS: N/R<br><br>Hemiplegia: 24 (Left= 7, Right= 17)	1- Time taken to perform 6 ADL tasks / Jebsen-Taylor Test of Hand Function [JTTHF]<br>2- Gross manual function / Box and blocks test<br>3- Bimanual used during play and ADL / Assisting Hand Assessment [AHA]	UMF and BMF	a. Stereognosis / Number of objects identified on 10<br>b. Two-point discrimination / Smallest distance in mm between two pressure points detected	Tactile	a. Stereognosis was associated with JTTHF (r= 0.7), Box and block (r= 0.69) and AHA (r= 0.74)<br><br>b. Two-point discrimination was associated with JTTHF (r=0.74), Box and block (0.76) and AHA (r= 0.76)<br><br>All significant
Guzzetta & al. (2007)	Adolescents and young adults (n=12) Age: Mean= 16.8; range 10-28y<br>Sex: 8M; 4W<br><br>Lesion type and/or timing: - Malformation of cortical development= 6<br>- Stroke= 3<br>- Periventricular leukomalacia = 3<br><br>- 6 before 24 weeks of gestation<br>- 3 between 24 and 36 weeks of gestation<br>- 2 term age<br>- 1 2 years old	MACS/GMFCS: N/R<br>Hemiplegia: 12 (Left=3, Right=9)	Quality of movements / Melbourne assessment of unilateral upper limb [MUUL]	UMF	All on a scale from 0 to 2<br><br>a. Tactile sense / Number of stimulations identified on different parts of the limbs with cotton<br>b. Proprioception / Recognition of slow displacement of finger<br>c. Graphaesthesia / Objects recognized on 5<br>d. Two-point discrimination / Number of numbers identified drawn on the palm of their hand<br>e.Double stimulation (DS) / Smallest distance in mm between two pressure points detected	Tactile and proprioception	No correlation identified between sensory and motor function at the whole group level. A significant correlation between motor and sensory function is present in the ipsilesional CST group ($p < 0.02$)
James & al. (2017)	Children and adolescents (n=37) Age: range: 4-15 years	Gross Motor Function Classification	1- Passive and active range of motion at elbow, forearm,	UMF and BMF	Stereognosis / Number of objects recognized on 12	Tactile	Positive significant correlation between stereognosis and AHA (r=0.54), but not with other

	Sex: 26M; 11W<br><br>Lesion type and/or timing: N/R	System levels 1 or 2<br><br>Hemiplegia: 37 (Left= 19; Right=18)	wrist, fingers and thumb levels<br>2- Bimanual used during play and ADL / Assisting Hand Assessment [AHA]<br>3- Gross manual / Box and blocks test<br>4- Shriners Hospitals Upper Extremity<br>Evaluation / [SHUEE]				upper limb function measurements.
James & al. (2015)	Children and adolescents (n=101) Age: Mean= 11 y and 8 months +/- 2y and 4 months<br>Sex: 51M; 50W<br><br>Lesion type and/or timing: N/R	MACS (%)<br>Level I= 24<br>Level II= 75<br>Level III= 1<br>GMFCS (%)<br>Level I= 45<br>Level II= 56<br><br>Hemiplegia: 101 (Left=48; Right=53)	1- Performance in functional tasks / Assessment of Motor and Process Skills [AMPS]<br>2- Time taken to perform 6 ADL tasks / Jebsen-Taylor Test of Hand Function [JTTHF]<br>3- Bimanual used during play and ADL / Assisting Hand Assessment [AHA]	UMF and BMF	Test of Visual Perceptual Skills, 3rd edition / a. Visual Discrimination; b. Visual Memory; c. Visual Spatial Relationships; d. Form Constancy; e. Visual Sequential Memory; f. Figure-Ground; g. Visual Closure. (TVPS-3)	Visual	AMPS motor sub form was associated with TVPS-3 (r=0.408; p<0.001), more specifically for Visual memory (r= 0.38, p < 0.001) and Visual Sequential memory (r= 0.39, p<0.001).
Kinnucan & al. (2010)	Children and adolescents (n=41) Age: Mean=8.7y; range 6-16y<br>Sex: 19M; 22W<br><br>Lesion type and/or timing: N/R	MACS/GMFCS: N/R<br><br>Hemiplegia: 34 (Left=18; right=23)<br>Triplegia: 7	Time taken to perform 6 ADL tasks / Jebsen-Taylor Test of Hand Function [JTTHF]	UMF	Stereognosis / Number of objects recognized on 12	Tactile	Stereognosis was associated with JTTHF (all p <0.04): Total score: -0.54; Cards: -0.39; Small objects: -0.52; Checkers: -0.60; Light objects: -0.33; Heavy objects: -0.48
Klingels & al. (2012)	Children and adolescents (n=81) Age: Mean= 9y 11 months +/- 3y 3 months; range 6-15y<br>Sex: 43M;38W	MACS<br>Level I= 29<br>Level II= 36<br>Level III= 16	1- Passive range of motion of shoulder, elbow and wrist<br>2-Muscle tone of shoulder, elbow and wrist / Modified Ashworth Scale	UMF and BMF	a. Two-point discrimination / Smallest distance in mm between two pressure points detected<br>b. Stereognosis / Number of objects recognized on 12	Tactile and Proprioception	a. Two-point discrimination was associated with MUUL (r= 0.66, p<0.0001), AHA (r=0.65, p<0.0001) and ABILHAND (r= 0.58, p<0.0001)

	Congenital = 69; acquired = 12	Hemiplegia: 81 (Left= 36, Right=45)	3- Muscle testing of shoulder, elbow and wrist<br>4-Grip strength / Jamar hydraulic hand Dynamometer<br>5- Quality of movements / Melbourne assessment of unilateral upper limb [MUUL]<br>6- Bimanual used during play and ADL / Assisting Hand Assessment [AHA]<br>7- Subjective manual performance / ABILHAND-Kids questionnaire		c. Tactile sense / Touch on the thumb, index and hand palm<br>d. Proprioception / Recognition of slow displacement of index (passive movement)		b. Stereognosis was associated with MUUL (r=0.72, p<0.0001), AHA (r= 0.63, p<0.001) and ABILHAND (r= 0.70; p<0.0001)<br>c. Tactile sense was associated with MUUL (r=0.39, p<0.01), and ABILHAND (r= 0.40, p<0.01) but not with AHA (r=0.38, p<0.01)<br>d. Proprioception was associated with MUUL (r=0.49, p<0.0001), AHA (r=0.4; p<0.0001) and ABILHAND (r= 0.38, p<0.01).<br><br>Wrist strength, stereognosis and proprioception predict MUUL score (respectively adding 68. 6 and 2% of variance); Wrist strength and grip strength predict AHA score (respectively adding 70 and 6% of variance); Wrist strength and stereognosis predict ABILHAND-Kids questionnaire (respectively adding 39 and 7% of variance).
Krumlinde-Sundholm & al. (2002)	<u>CP subjects:</u><br><br>Children and adolescents (n=25) Age: range 8-15y	MACS/GMFCS: N/R<br><br>Hemiplegia: 25 (Left=11, Right= 14)	1- Pick-up test n / Time in seconds to pick out 10 wooden cubes from a box (Dexterity)<br>2- Bimanual tasks / Performance in eight everyday tasks	UMF and BMF	a. Tactile pressure detection / Force required to bend the thinnest monofilament detected by child (Semmes-Weinstein monofilaments)	Tactile and Visual	a. Tactile pressure detection was associated with pick-up test (r=-0.4) but not bimanual tasks (r=-0.13)<br>b. Two-point discrimination-3mm was associated with pick-

	Lesion type and/or timing: N/R<br><br>Control subjects:<br><br>Typically developing adolescents and children (n=19) Age: Range 5-18y				b. Two-point discrimination / Smallest distance in mm between two pressure points detected (3mm and 7 mm tested)<br>c. Stereognosis / Number of objects recognized on 6 familiar objects and 10 forms (familiar object and abstract forms)		up test (r=-0.6) but not with bimanual tasks (r=0.25). Two-point discrimination-7mm was associated with pick-up test (r=-0.71) and bimanual tasks (r=0.41)<br>c. Stereognosis of familiar object was associated with pick-up test (r= -0.65) but not with bimanual tasks (r=0.36). Stereognosis of forms was associated with pick-up test (r= -0.60) but not with bimanual tasks (r=0.35)
Kuczynski & al. (2016)	CP subjects:<br><br>Adolescents (n=40) Age: AIS: Mean= 13.0 +/- 3.6y; PVI : Mean= 12.1 +/- 3.5y<br>Sex :<br>AIS : 12M; 10W<br>PVI : 11M; 7W<br><br>22 Arterial ischemic stroke (AIS) and 18 periventricular venous infarctions (PVI)<br><br>Control subjects:	MACS<br>AIS<br>Level I : 6<br>Level II : 12<br>PVI<br>Level I : 8<br>Level II :5<br><br>Hemiplegia: 40<br>AIS: Left= 8, Right=14 PVI: Left=9; Right=9	1- Bimanual used during play and ADL / Assisting Hand Assessment [AHA]<br>2- Upper limb motor recovery on a scale of 1 to 7 / Chedoke McMaster Stroke Asssessment [CMSA]<br>3- Fine motor function / Purdue Pegboard test	UMF and BMF	a. Arm position matching test / 1. Variability, contraction/expansion; 2. Systematic shift with and without vision (Variability, shiftxy) (KINARM)	Proprioception	AHA scores were negatively correlated with Variability (no vision) in AIS ($\varrho = -0.64$, $P < 0.01$) but not PVI ($\varrho = 0.12$, $P = 0.7$). AHA scores correlated with Shiftxy(vision) in AIS ($\varrho = -0.49$, $P < 0.05$) but not PVI ($\varrho = 0.20$,<br>$P = 0 .5$).<br><br>Fine motor function as determined by the PPB was inversely correlated with both Varxy ($\varrho = -0.51$, $P < 0.01$) and Shiftxy ($\varrho = -0.52$, $P < 0.01$) in AIS but not PVI or controls.

Kurtaran & al. (2015)	Adolescents (n=60) Age: Mean = 12.3 +/- 3.6y<br>Sex: 38M;22W<br><u>CP subjects:</u><br><br>Children (n=36) Age: Mean= 6.28 +/- 1.95y<br>Sex: 66%M; 24%W<br><br>Lesion type and/or timing: N/R<br><br><u>Control subjects:</u><br><br>Children (n=18) Age: Mean= 6.61 +/- 1.61y	MACS/GMFCS: N/R<br><br>Hemiplegia: 6; Diplegia: 13; Quadriplegia: 17	Functional status / On a scale from 1 (no limitation) to 4 (impossible to perform the task)	BMF (subjective)	Stereognosis / Number of objects recognized on 10	Tactile	Significant correlation ($p<0.05$) between functional level and stereognosis.
Law & al. (2008)	Children, adolescents and adults (n=30) Age: Mean= 12.48y; range 6-33y<br><br>Lesion type and/or timing: N/R	MACS/GMFCS: N/R<br><br>Hemiplegia: 9; Diplegia: 8; Triplegia: 3; Quadriplegia: 10	1- Quality of movements based / Melbourne assessment of unilateral upper limb [MUUL]<br>2- Grasping / Functional Hand Grip<br>Test [FHGT]<br>3- Function / Functional Independence Measure for Children [WeeFIM]	UMF	a. Stereognosis / Number of objects recognized on 13<br>b. Two-point discrimination wo points discrimination / Smallest distance in mm between two pressure points detected	Tactile	Sensory deficits showed a statistically significant correlation with the MUUL, but not with the FHGT.<br>1- MUUL- two-point discrimination: $r=0.422$ ($p = 0.002$)<br><br>2- MUUL-stereognosis: $r= 0.440$ ($p = 0.036$).<br><br>3- WeeFIM- two-point discrimination: $r= -0.519$, $p=0.011$
O'Malley & al. (1977)	Children and adolescents (n=18) Age: Mean= 9y 5	MACS/GMFCS: N/R	Fine motor skills and Bilateral integration /The Beery Developmental Test	UMF and BMF	a. Stereognosis<br>b. Graphesthesia<br>c. Tactile localization	Visual, Tactile	Bilateral integration was not associated to any of the sensory function and:

	months; range 5y 8months- 15y 3 months<br><br>15 perinatal injury and 3 acquired, post natal injury	Hemiplegia: 18 (Left= 9; Right=9)			d. Double simultaneous stimulation<br>e. Touch<br>f. Visuo-spatial perceptual skills<br>g. Visuo-spatial constructive skills<br><br>Not detailed (The Beery Developmental Test of<br>Visual Motor Integration and the Ayres<br>battery or Southern California Sensory)		a. r= 0.1, p= 0.693<br>b. r= 0.055, p =0.827<br>c. r= 0.025, p=0.925<br>d. r=0.06, p=0.814<br>e. r=0.395, p= 0.104<br>f. r= 0.204, p= 0.417<br>g. r= 0.204, p =0.417
Robert & al. (2013)	Children (n=16) Age: Mean= 8.6y; range 6-11y<br>Sex: 7M; 9W<br><br>Lesion type and/or timing: N/R	MACS<br>Level II: 7<br>Level III: 5<br>Level IV: 4<br><br>Hemiplegia: 16 (Left= 9; Right= 7)	1- Upper limb passive range of motion (ROM)<br>2- Quality of movements based / Melbourne assessment of unilateral upper limb [MUUL]	UMF	a. Stereognosis / number of objects recognized in %<br>b. Touch / Light touch-measure on a total of 20<br>c. Proprioception/ Level of impairment on a scale of 8	Tactile and proprioception	a. Stereognosis was association with MUUL (r= 0.705, p= 0.015) but not with ROM (r=0.451, p= 0.08)<br>b. Light touch was not associated with ROM (r=0.004, p=0.988) and MUUL (r= -0.598, p= 0.52)<br>c. Proprioception was associated with ROM (r= 0.529; p=0.035) and MUUL (r=0.656, p= 0.028)
Russo & al (2019)	Children and adolescents (n=108) Age: Mean= 8y 7 months +/- 3y 9 months; range 3-16y<br>Sex: 62M; 46W<br><br>Lesion type and/or timing: N/R	GMFCS<br>Level I=86<br>Level II=15<br>Levels III–<br>V=7<br><br>Hemiplegia: 108 (Left= 49; Right= 59)	1- Performance in functional tasks / Assessment of Motor and Process Skills [AMPS]<br>2- Functional capacity / Pediatric Evaluation of Disability Inventory [PEDI]<br>3- Subjective bimanual function (CP Register Assessment of	UMF and BMF	a. Stereognosis / Number of objects recognized on 10<br>b. Tactile sense / Number of fingers identified when touch<br>c. Two-point discrimination / Yes/no identification of 8 mm distance	Tactile	Sensation had a significant positive direct effect on manual ability (b=0.52) and a positive indirect effect for sensory function<br>(b=0.24) on independence self-care.

			Bimanual Upper Limb Function)<br>4- Ordinal scale of unimanual capacity (manual ability, The South<br>Australian Cerebral Palsy Register classification)		d. Graphesthesia / Number of letters recognized on 6 when drawn on the palm of the hand		
Sakzewski & al. (2010)	<u>CP subjects:</u><br><br>Children and adolescents (n=70) Age: Mean= 10y 6 months +/- 3y; range 5-17y<br>Sex: 39M; 31W<br><br>Lesion type and/or timing: N/R<br><br><u>Control subjects:</u><br><br>Typically developing children and adolescents(n=15) Age: Mean 8y 8months +/- 2y 7months; range 5–16y<br>Sex: 7M;8W	MACS<br>Level I: 18 Level II: 51<br>Level III:1<br>GMFCS<br>Level I:18<br>Level II: 52<br><br>Hemiplegia: 70 (Left = 30; Right=40)	1- Quality of movements based on 16 items / Melbourne assessment of unilateral upper limb [MUUL]<br>2- Time taken to perform 6 ADL tasks / Jebsen-Taylor Test of Hand Function [JTTHF]<br>3- Bimanual used during play and ADL / Assisting Hand Assessment [AHA]<br>4- Grip strength	UMF and BMF	a. Two-point discrimination / Smallest distance in mm between two pressure points detected<br>b. Stereognosis / Number of objects recognized on 6	Tactile	a. Two-point discrimination was associated with Grip strength (r= -0.38), JTTHF (r= 0.63), MUUL (r= -0.54) and AHA (r = -0.57)<br>b. Stereognosis was associated with Grip strength (r= 0.31), JTTHF (r= -0.64), MUUL (r= 0.53) and AHA (r= 0.68) a.- MUUL: r= -0.54<br><br>All significant<br><br>- MUUL and stereognosis explains 75.1 % of AHA variance<br>- MUUL and stereognosis explains 75.1 % of AHA variance<br>- Stereognosis explains 73.7 % of JTHFT variance<br>- Two-point discrimination explains 73.6 % of MUUL variance

Simon-Martinez & al. (2018)	Children and adolescents (n=75) Age: Mean= 11y 1month +/- 3y 6 months<br>Sex: 42M; 33W<br><br>PV: Periventricular=34; CSC: Corticosubcortical=18	MACS<br>Level I=25<br>Level II= 25<br>Level III= 25<br><br>Hemiplegia: 75 (Left=39; Right= 36)	1- Time taken to perform 6 ADL tasks / Jebsen-Taylor Test of Hand Function [JTTHF]<br>2- Bimanual used during play and ADL / Assisting Hand Assessment [AHA]<br>3- Grip strength / Jamar hydraulic hand dynamometer	UMF and BMF	a. Two-point discrimination / Smallest distance in mm between two pressure points detected<br>b. Stereognosis / Number of objects recognized on 6<br>c. Tactile pressure detection / Force required to bend the thinnest monofilament detected by child (Semmes-Weinstein monofilaments)	Tactile	a. Two-point discrimination was not associated with any of motor tasks.<br>b. Stereognosis was associated with grip strength ratio (r = −0.60, p < 0.001), JTHFT ratio (r = −0.60, p < 0.001) and AHA (r = 0.61, p < 0.001)<br>c. Tactile pressure detection was not associated with any of motor tasks.
Surkar & al. (2018)	<u>CP subjects:</u><br><br>Children (n=13) Age: Mean = 6.8y +/- 2.9y<br>Sex: 7M;6W<br><br>Periventricular leukomalacia= 2, Perinatal stroke= 9; Neonatal stroke= 1; Schizencephaly =1<br><br><u>Control subjects:</u><br><br>Typically developing children (n=15) Age: Mean= 5.8y +/- 1.1y<br>Sex: 8M;7W	MACS<br>Level II: 2<br>Level III: 9<br>Level IV:2<br><br>Hemiplegia: 13 (Left= 8; Right= 5)	Reach and grasp 1- Eye-hand coordination / a. Movement Onset Asynchrony: Time lag between the first gaze to the starting stimulus and hand initiation.<br>b. Movement Termination Asynchrony: Time lag between the object placement and gaze at the target.<br>c. Frequency of gaze shift: Number of times the gaze moved in each sequence of movement.<br><br>2- Action planning / a. Reaction Time: Time lag between the first gaze to the starting stimulus and hand initiation.<br>3- Action execution / a. Movement time: Time to	UMF	Visual anticipatory pattern / Time lag between appearance of a starting stimulus and first gaze at the stimulus	Visual	- CTRL (341.12 + 82.18 ms) = faster anticipatory gaze time than HCP (878.44 + 76.58 ms)<br>- CTRL (250.17 + 115.90 ms) = shorter latency between gaze timing and hand initiation than HCP (764.81 + 107.99 ms)<br>-CTRL (121.03 + 51.11 ms)= shorter latency between the gaze leaving the fixation target and starting to move toward the final target than HCP (-220.19 + 56.60 ms)<br>- CTRL (189.03 + 45.66 ms)= quicker and shorter anticipatory gaze time than HCP (356.10 + 51.1 ms).<br>- CTRL (591.66 + 606.79 ms)= shorter reaction time than HCP (2579.87 + 565.35 ms).

			complete each movement sequence				CTRL (640.19 + 92.26 ms) = shorter movement time than HCP for two different sequences of movements
Van Roon & al. (2005)	CP subjects:<br><br>Adolescents and adults (n=8) Age: Mean= 18y =/- 2y 1 month; range 15y 1 month- 20y 6 months<br>Sex: 5M;3W<br><br>Lesion type and/or timing: N/R<br><br>Control subjects:<br><br>Adults (n=8) Age: Mean= 22y 5 months +/- 2y 11 months; range 19y 2months- 28y 5 months	MACS/GMFCS: N/R<br><br>Tetraplegia: 8	Straight line- drawing movements / 1- Lateral deviation (LD) from straight line between starting point and midpoint of target; 2- Percentage of movement time outside de lines (% MT); 3- Axial force (AF); 4- Movement time (MT); 5- Dysfluency (D)	UMF	Proprioception / Passive and active proprioception	Proprioception	All significant<br>Visual<br>1 (LD)- With vision: CP absolute deviations > CTRL absolute deviations ($p < 0.05$).<br>Proprioception:<br>Active proprioception error high = >LD ($r = 0.89$, $p = 0.004$) and shorter MTs ($r = –0.86$, $p = 0.014$)<br>Passive proprioception error high = > variability for LD ($r = 0.64$, $p = 0.043$)<br>AF > higher absolute error scores in the passive proprioception test ($r = 0.86$, $p = 0.007$), higher variability of the error in the passive proprioception test ($r = 0.64$, $p = 0.043$) and<br>higher variability of the error in the active proprioception test ($r = 0.74$, $p = 0.019$)
Woodward & al. (2019)	CP subjects:<br><br>Children and adolescents (n=17) Age: Median=	MACS/GMFCS: N/R<br><br>Hemiplegia: 17; (Left= 9; Right= 8)	1- Quality of movements based on 16 items Melbourne assessment of unilateral upper limb [MUUL]	UMF and BMF	Robotic matching limb / Spatial variability from the expected limb position (KINARM)	Proprioception	MUUL -proprioception : $r = 0.075$; AHA- proprioception: $r = 0.312$<br><br>Not significant

	11.3y; interquartile range 9.1-14.5y<br>Sex: 64.7%M; 35.3%W<br><br>Periventricular venous infarction (PVI)<br><br><u>Control subjects:</u><br><br>Children and adolescents (n=21) Age: Median= 12.6y; interquartile range 9.5-15.5y	2- Bimanual used during play and ADL / Assisting Hand Assessment [AHA]	

Legend :

MACS : Manual Ability Classification System; Gross Motor Function Classification System; UMF: Unilateral motor function; BMF: Bilateral motor function; r: correlation coefficient; CP: cerebral palsy (Y: young; A: adult); y: years; M: man; W: woman; N/R: not reported; N/A: not applicable; ADL: activity of daily living; MCP: metacarpophalangeal; DH: dominant hand; NDH: non dominant hand; MA: manual ability; MP: subjective manual performance; MUUL: Melbourne Unilateral Upper Limb Assessment; JTTHF: Jebsen-Taylor Test of Hand Function; AHA: Assisting hand assessment; DVPT-2: Developmental Test of Visual Perception 2nd ed; MVPT-R: Motor-Free Visual Perceptual Test Revised ; PQ: Perceptual quotient; GVPQ: General visual perception quotient; MRPQ: motor reduced perception quotient; VMI: Visual motor integration; PVL: periventricular leukomalacia; DQ: developmental quotient; HCP: Hemiplegia cerebral palsy type; DCP: Diplegia cerebral palsy type; CST: corticospinal tract; SP: spastic; ATH: athetoid; SHUEE: Shriners Hospitals Upper Extremity Evaluation; TVPS-3: Test of Visual Perceptual Skills, 3rd edition; AMPS: Assessment of Motor and Process Skills; mm: millimeters; AIS: Arterial ischemic stroke; PVI: periventricular venous infarction; RL: Response latency; IDE: Initial direction error; PSR: Peak speed ratio; CTRL: control; FGHT: Functional Hand GripTest; WeeFIM: Functional Independence Measure for Children; ROM: range of motion; CSC: Corticosubcortical; ms : milliseconds; MT: movement time; AF: axial force; D: dysfluency; cm: centimeter; LD: lateral deviation

brain sciences

MDPI

Table S3: Assessment of study quality by an appraisal quality tool (Kmet d & al., 2004) 1

Study	Q1	Q2	Q3	Q4	Q8	Q9	Q10	Q11	Q12	Q13	Q14	Total	%	Quality
Alves-Pinto & al. (2017)	2	2	1	2	2	1	2	2	1	2	2	19	86	High
Arnould & al. (2007)	2	2	1	2	2	2	2	2	2	2	2	21	95	Very high
Arnould & al. (2014)	2	1	1	2	1	2	1	1	2	2	2	17	77	Moderate
Auld & al. (2012)	2	2	2	1	2	2	2	1	1	2	2	19	86	High
Bleyenfeult & al. (2011)	2	1	1	2	1	1	2	0	0	2	2	14	63	Low
Bumin &al. (2010)	2	1	2	1	2	2	1	2	1	2	2	18	81	High
Burtner & al. (2006)	2	2	1	2	2	2	2	2	1	2	2	20	90	Very high
Cioni & al. (2000)	2	2	2	1	1	2	2	0	1	2	2	17	77	Moderate
Cooper & al. (1995)	2	2	1	1	1	1	1	2	1	2	2	16	72	Moderate
De Campos & al. (2014)	2	1	1	2	2	1	2	1	2	2	2	18	81	High
Duque & al. (2003)	2	1	1	2	2	2	2	0	2	2	2	18	81	High
Eliasson & al. (1995)	2	1	1	0	1	1	2	1	2	2	2	15	68	Low
Gordon & al. (2006)	2	1	1	2	1	2	2	1	2	2	2	18	81	High
Gordon & al. (1999)	2	1	2	0	1	2	2	0	2	2	2	15	68	Moderate
Guedin & al (2018)	2	1	2	2	1	2	0	2	2	2	2	18	81	High
Gupta & al. (2017)	2	1	1	2	1	2	2	1	1	2	2	17	77	Moderate
Guzzetta & al. (2007)	2	1	1	2	1	1	1	0	2	2	2	15	68	Low
James & al. (2017)	2	1	1	0	2	2	1	2	1	2	2	16	72	Moderate
James & al (2015)	2	2	2	2	1	2	1	1	1	2	2	18	81	High
Kinnucan & al. (2010)	2	1	1	2	1	2	1	0	1	1	2	14	63	Low
Klingels & al. (2012)	2	1	1	2	2	2	2	2	2	2	2	20	90	Very high
Krumlinde-Sundholm	2	1	2	1	1	2	1	1	2	2	2	17	77	Moderate

& al. (2002)														
Kuczynski & al. (2016)	2	1	2	2	2	2	2	2	2	2	2	21	95	Very high
Kurtaran & al. (2015)	1	1	1	0	1	2	1	2	1	1	2	13	59	Very low
Law & al. (2008)	1	1	2	1	1	2	1	0	1	1	2	13	59	Very low
O'Malley & al. (1977)	1	2	1	1	1	2	0	0	1	1	2	12	54	Very low
Robert & al. (2013)	2	2	2	2	1	2	2	1	1	2	2	19	86	High
Russo & al. (2019)	2	2	2	2	1	2	2	1	1	2	2	20	90	Very high
Sakzewski & al. (2010)	2	2	0	2	2	2	2	1	2	2	2	19	86	High
Simon-Martinez & al. (2018)	2	1	2	2	2	2	2	1	1	2	2	19	86	High
Surkar & al. (2018)	2	1	2	2	2	1	2	2	1	2	2	19	86	High
Van Roon & al. (2005)	2	1	1	2	2	1	2	2	1	2	2	18	81	High
Woodward & al. (2019)	2	1	2	2	2	2	2	2	1	2	2	20	90	Very high

Legend: Design requirement: Q1: Question/objective sufficiently described?; Q2: Study design evident and appropriate?; Q3: Method of subject/comparison group selection or source of information/input variables described and appropriate?; Q4: Subject (and comparison group, if applicable) characteristics sufficiently described?; Q8: Outcome and (if applicable) exposure measure(s) well defined and robust to measurement / misclassification bias? Means of assessment reported?; Q9: Sample size appropriate?; Q10: Analytic methods described/justified and appropriate?; Q11: Some estimate of variance is reported for the main results?; Q12: Controlled for confounding?; Q13: Results reported in sufficient detail?; Q14: Conclusions supported by the results?
